# Diagnostic, Therapeutic, and Prognostic Value of the Thrombospondin Family in Gastric Cancer

**DOI:** 10.3389/fmolb.2021.647095

**Published:** 2021-04-28

**Authors:** Yi Lu, Xianhe Kong, Weijie Zhong, Minhui Hu, Chujun Li

**Affiliations:** ^1^ Department of Gastrointestinal Endoscopy, The Sixth Affiliated Hospital, Sun Yat-sen University, Guangzhou, China; ^2^ Guangdong Provincial Key Laboratory of Colorectal and Pelvic Floor Diseases, The Sixth Affiliated Hospital, Sun Yat-sen University, Guangzhou, China

**Keywords:** gastric cancer, THBS1, THBS2, THBS3, THBS4, cartilage oligomeric matrix protein

## Abstract

**Background:** Gastric cancer (GC) is the fifth leading cancer in the world. The dysregulated expressions of the thrombospondin (THBS) family were reported to associate with GC, but their relations with tumor stage, prognosis, and correlations with tumor immunity have not been systematically reported.

**Methods:** We used versatile public databases such as Oncomine, GEPIA, UALCAN, Kaplan–Meier Plotter, LinkedOmics, STRING, cBioPortal, TIMER, and TISIDB to analyze the expression and mutations of different *THBSs* in GC, along with their functional networks, survival analysis, and tumor–immune interactions.

**Results:** The mRNA levels of *THBS2*, *THBS4,* and *COMP* were significantly higher in the tumor tissues; the expression levels of *THBS1*, *THBS2*, and *THBS4* were higher in stages 2–4 than that of stage 1; patients with high expression of *THBS1*, *THBS2*, *THBS4*, and *COMP* had poor OS; the genes correlated with *THBSs* were enriched in focal adhesion, glycosaminoglycan biosynthesis, ECM-receptor interaction, and hedgehog signaling pathway; *THBS1* and *THBS4* expression had significant correlations with tumor purity, and all the *THBSs* expression correlated with macrophage and dendritic cells infiltration.

**Conclusions:** THBS2, THBS4, and COMP were potentially diagnostic markers for GC; THBS1, THBS2, THBS4, and COMP were potentially prognostic markers for GC; investigating the relations of THBSs and tumor immunology might help in immunotherapy of GC, while more studies are needed to confirm these results.

## Introduction

Gastric cancer (GC) ranks as the fifth leading cancer and the third leading cause of cancer-related death globally, presenting as a significant public health problem, especially in Asian areas (Petryszyn et al., 2020). It was estimated that the incidence of GC was 1,033,000 globally in 2018 and that the GC-related deaths were 783,000 (Bray et al., 2018). Although we have achieved considerable advancements in diagnostic and therapeutic methods in GC, the 5-year survival rate of advanced GC is still not that satisfactory, which is reported to be 18–29% (Banks et al., 2019). Therefore, more effective potential drug targets and prognostic biomarkers should be identified.

The thrombospondin (THBS) family is of extracellular matrix (ECM) proteins, which can be classified into two groups based on their molecular architecture. The first group consists of two trimeric proteins [thrombospondin 1 (THBS1) and thrombospondin 2 (THBS2)], and the second one includes pentameric proteins [thrombospondin 3 (THBS3), thrombospondin 4 (THBS4), and cartilage oligomeric matrix protein (COMP)] (Sid et al., 2004). The THBSs affect multiple biological processes involving tissue remodeling, angiogenesis, and neoplasia, and the mechanisms are extremely complicated (Lawler and Detmar, 2004). In the early stage of cancer progression, the normal tissues secrete THBS1 and THBS2, playing a role as an antiangiogenic fence, while under some circumstances, they might switch to an angiogenic phenotype, acting as supporter for tumor development and metastasis, and their roles in GC were not consistent in different studies (Albo et al., 2002; Kazerounian et al., 2008; Sun et al., 2014; Eto et al., 2015; Ao et al., 2018). The relationship between THBS3 and GC has not been reported up to now, and in osteosarcoma, THBS3 was found to express at significantly high levels in patients with metastasis (Dalla-Torre et al., 2006). High expression of THBS4 and COMP hypomethylation was reported to correlate with poor prognosis (Chen et al., 2019; Liang et al., 2019). To our knowledge, the dysregulated expression of the THBS family and their relations with tumor stage, prognosis, and correlations with tumor immunity in GC have not been systematically reported. With the revolutionized development of microarray and bioinformatic technology, we conducted this study using the data from The Cancer Genome Atlas (TCGA) and other versatile public databases to analyze the expression levels and mutations of different *THBSs* in GC, along with their functional networks, prognostic values, and tumor–immune interactions, so as to reveal potential diagnostic, therapeutic, and prognostic targets for GC, and the results in different databases were verified with each other to make the results more convincible.

## Materials and Methods

### Oncomine Database Analysis

We used the Oncomine database version 4.5 (www.oncomine.org) to determine the mRNA levels and DNA copy numbers of *THBSs* in patients with GC. Oncomine, which involves 715 datasets and 86,733 samples, is a cancer microarray database uncovering the complex gene expression patterns of a variety of cancers (Rhodes et al., 2004). The cutoff criteria were set as gene rank top 10%, fold change >2, and *p* <0.05. As there were several datasets comparing the mRNA expression levels and DNA copy numbers of *THBSs* between tumor and normal tissues (Chen et al., 2003; Cho et al., 2011; Cui et al., 2011; Deng et al., 2012; D'Errico et al., 2009; Wang et al., 2012), Oncomine was capable of pooling the results together, and the results were shown as heat maps.

### Gene Expression Profiling Interactive Analysis

GEPIA (http://gepia.cancer-pku.cn/) is a gene expression analysis web which contains 9,736 tumors and 8,587 normal samples from the TCGA and the GTEx databases (Tang et al., 2017). It is equipped with the functions of differential expression analysis, stage analysis, survival analysis, multiple gene comparison, similar gene detection, and so forth (Tang et al., 2017). Here we used GEPIA to compare the expression levels and its relationship with GC stages. The results were expressed as boxplots and violin plots, and the cutoff criteria were set as *p <* 0.05 and |Log2FC| > 1.

### UALCAN Analysis

UALCAN (http://ualcan.path.uab.edu/) obtains and processes the gene expression and patient’s clinical data from TCGA and generates differential expression, survival analysis, methylation information, and the like. Furthermore, it can compare the differential expression levels in various subgroups (by race, gender, stage, etc.). Here, we used UALCAN to verify the comparison results of expression levels of *THBSs* and their relationship with tumor stages.

### Survival Analysis

Kaplan–Meier Plotter (http://kmplot.com/analysis/) includes data sources from European Genome-phenome Archive (EGA), Gene Expression Omnibus (GEO), and TCGA, which is capable of assessing the survival results of 21 types of cancer including GC (Szász et al., 2016). We used it to perform the overall survival (OS) analysis. The split cutoff of low and high expression was set in auto select best cutoff model, and biased arrays were excluded. The log-rank test was used for computing *p*-value, and *p* < 0.05 was regarded as significant. Hazard ratio (HR), 95% confidence interval (CI), and false discovery rate (FDR) were also generalized.

### LinkedOmics Database Analysis

LinkedOmics (http://www.linkedomics.org/) contains multi-omics and clinical data for 32 types of cancer from TCGA (SV et al., 2018). The Spearman correlation test was applied to find the significantly associated genes. The LinkFinder module showed the association result, presenting as tables, heat maps, and volcano plots. The LinkInterpreter module could perform the Gene Set Enrichment Analysis (GSEA), such as Gene Ontology (GO) and Kyoto Encyclopedia of Genes and Genomes (KEGG) pathways, and the former includes biological process (BP), cellular component (CC), and molecular function (MF). The selection criteria were *p* < 0.05 and 500 simulations.

### Protein–Protein Interaction Network Analysis

We put the top 20 positively and top 20 negatively associated genes of each *THBSs* to the STRING database version 11.0 (website: http://string-db.org/) to obtain the information of PPI (Szklarczyk et al., 2017). The cutoff criterion was set as combined score >0.4. Then, we used Cytoscape version 3.6.0 to picture the interaction networks of the correlated genes (Shannon et al., 2003). Genes with node degree ≥10 were regarded as potential hub genes. MCODE plug-in was applied to find the significant cluster, and two clusters were found. The elements in the two clusters were all among the potential hub genes.

### cBio Cancer Genomics Portal (cBioPortal) Analysis

cBioPortal (https://www.cbioportal.org/) converts molecular information of cancer tissues and cell lines into genetic, epigenetic, and gene expression data (Cerami et al., 2012; Gao et al., 2013). We used it to figure out the *THBSs* alterations in GC (stomach adenocarcinoma, TCGA, and Firehose Legacy were chosen). We also estimated the mutual correlations of *THBSs* by analyzing their mRNA expression (RNA Seq V2 RSEM), and then the Spearman correlation coefficient was put into Microsoft Excel 2007 to draw the heat maps.

### Tumor Immunology Analysis

Tumor Immune Estimation Resource (TIMER, https://cistrome.shinyapps.io/timer/) can analyze immune infiltrates in a diversity of cancers systematically (Li et al., 2017). We used it to explore the associations between gene expression, survival outcome, somatic copy number alterations (CNA), and immune infiltration. TISIDB (http://cis.hku.hk/TISIDB/) is another web portal to analyze tumor and immune system interaction. It integrates multiple data types, and users can explore the correlations of a certain gene with tumor-infiltrating lymphocytes, immunomodulators, chemokines, subtypes, and survival information (Ru et al., 2019).

## Results

### Transcriptional Levels of *THBSs* in Patients With Gastric Cancer

**FIGURE 1:**
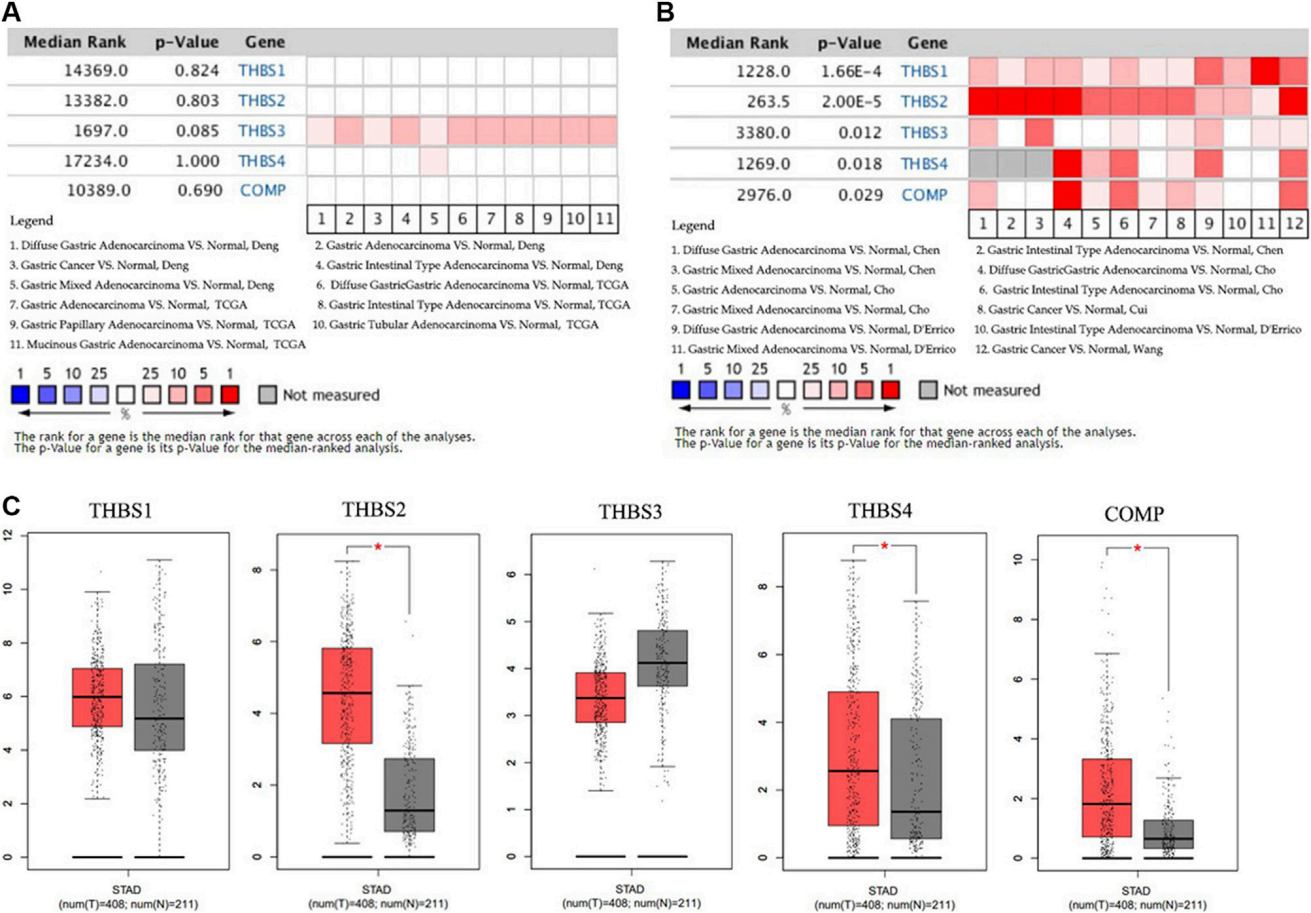
Heat maps showing variations between the gastric cancer tumor tissues and normal tissues of **(A)** the DNA copy numbers of the *THBSs* and **(B)** the mRNA expression levels of the *THBSs* (Oncomine); **(C)** boxplot showing the expression levels of *THBSs* in gastric cancer (red star means *p* < 0.05, GEPIA).

Data in the Oncomine database showed that the DNA copy numbers of all the *THBSs* in the tumor tissues were not statistically different from the normal (Figure 1A), but the mRNA expression levels of the all the *THBSs* were significantly higher in the tumor tissues; moreover, the levels were associated with the cancer histopathologic types (Figure 1B). The boxplot results in the GEPIA showed that the expression levels of *THBS2*, *THBS4*, and *COMP* were significantly higher in the GC tumor tissues than the normal tissues, while the expression levels of *THBS1* and *THBS3* were not significantly different (Figure 1C). Results from the UALCAN database also indicated that the expression levels of *THBS1* were not significantly different between the tumor and normal tissues, while for other *THBSs* certain differences existed and which might also be associated with tumor stages (Table 1).

**TABLE 1:** The UALCAN results of the differential *THBSs* expression levels between the gastric cancer and normal tissues and correlation with tumor stage.

Expression	*p*-value	Stage 1	Stage 2	Stage 3	Stage 4
THBS1	Normal	0.27	0.19	0.12	0.11
	Stage 1	—	5.36E-3	2.50E-3	6.28E-3
THBS2	Normal	0.14	1.62E-12	1.62E-12	2.00E-7
	Stage 1	—	3.92E-9	4.78E-9	1.90E-5
THBS3	Normal	0.77	0.02	0.04	0.11
	Stage 1	—	0.14	0.20	0.27
THBS4	Normal	0.017	6.68E-3	1.74E-3	0.11
	Stage 1	—	4.79E-7	3.78E-10	4.12 E-3
COMP	Normal	0.27	0.025	1.46 E-3	4.05 E-5
	Stage 1	—	0.20	0.09	0.87

### Relationship Between the THBSs and Tumor Stage in Gastric Cancer

**FIGURE 2:**
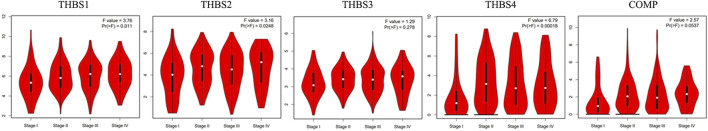
Stage plot showing the correlation between *THBSs* expression with tumor stage in gastric cancer (GEPIA).

Stage plot in the GEPIA showed that the expression levels of *THBS1*, *THBS2*, and *THBS4* varied with tumor stages of GC, while no significant variation was found in *THBS3* and *THBS5* (Figure 2). Furthermore, analysis from UALCAN also supported that the expression levels of *THBS1*, *THBS2*, and *THBS4* were higher in stages 2–4 than that of stage 1 and that the expression levels of *THBS3* and *COMP* did not vary with different tumor stages (Table1).

### THBSs and Survival Analysis

**FIGURE 3:**
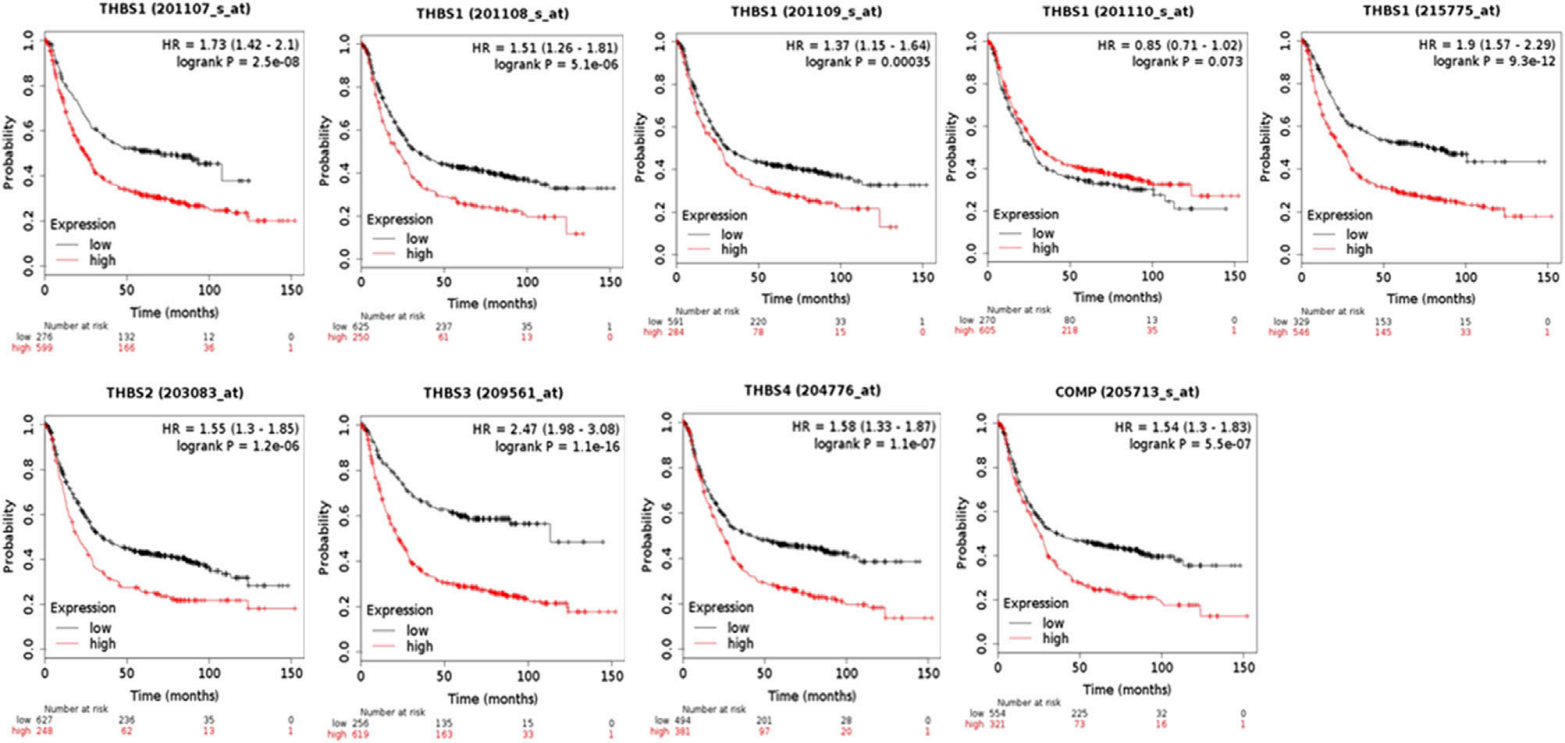
The overall survival analysis of the patients with gastric cancer grouped by expression levels of *THBSs* (Kaplan–Meier plotter).

When using the Kaplan–Meier Plotter for survival analysis, THBS1 had five probe IDs, and the rest THBSs each had only one. The probe IDs of 201107_s_at (HR = 1.73, 95% CI: 1.42–2.1, *p* = 2.5E-8, FDR 1%), 201108_s_at (HR = 1.51, 95% CI: 1.26–1.81, *p* = 5.1E-6, FDR 1%), 201109_s_at (HR = 1.37, 95% CI: 1.15–1.64, *p* = 3.5E-4, FDR 10%), and 215775 _at (HR = 1.9, 95% CI: 1.57–2.29, *p* = 9.3E-12, FDR 1%) suggested that high expression of *THBS1* had poor OS, while the probe IDs of 201110_s_at (HR = 0.85, 95% CI: 0.71–1.02, *p* = 0.07, FDR 100%) did not. In addition, high expression of *THBS2* (HR = 1.55, 95% CI: 1.3–1.85, *p* = 1.2E-6, FDR 1%), *THBS3*(HR = 2.47, 95% CI: 1.98–3.08, *p* = 1.1E-16, FDR 1%), *THBS4* (HR = 1.58, 95% CI: 1.33–1.87, *p* = 1.1E-7, FDR 1%), and *COMP* (HR = 1.54, 95% CI: 1.3–1.83, *p* = 5.5E-7, FDR 1%) were all correlated with poor OS (Figure 3).

### Co-Expression Genes Correlated With *THBSs* in Gastric Cancer

**FIGURE 4:**
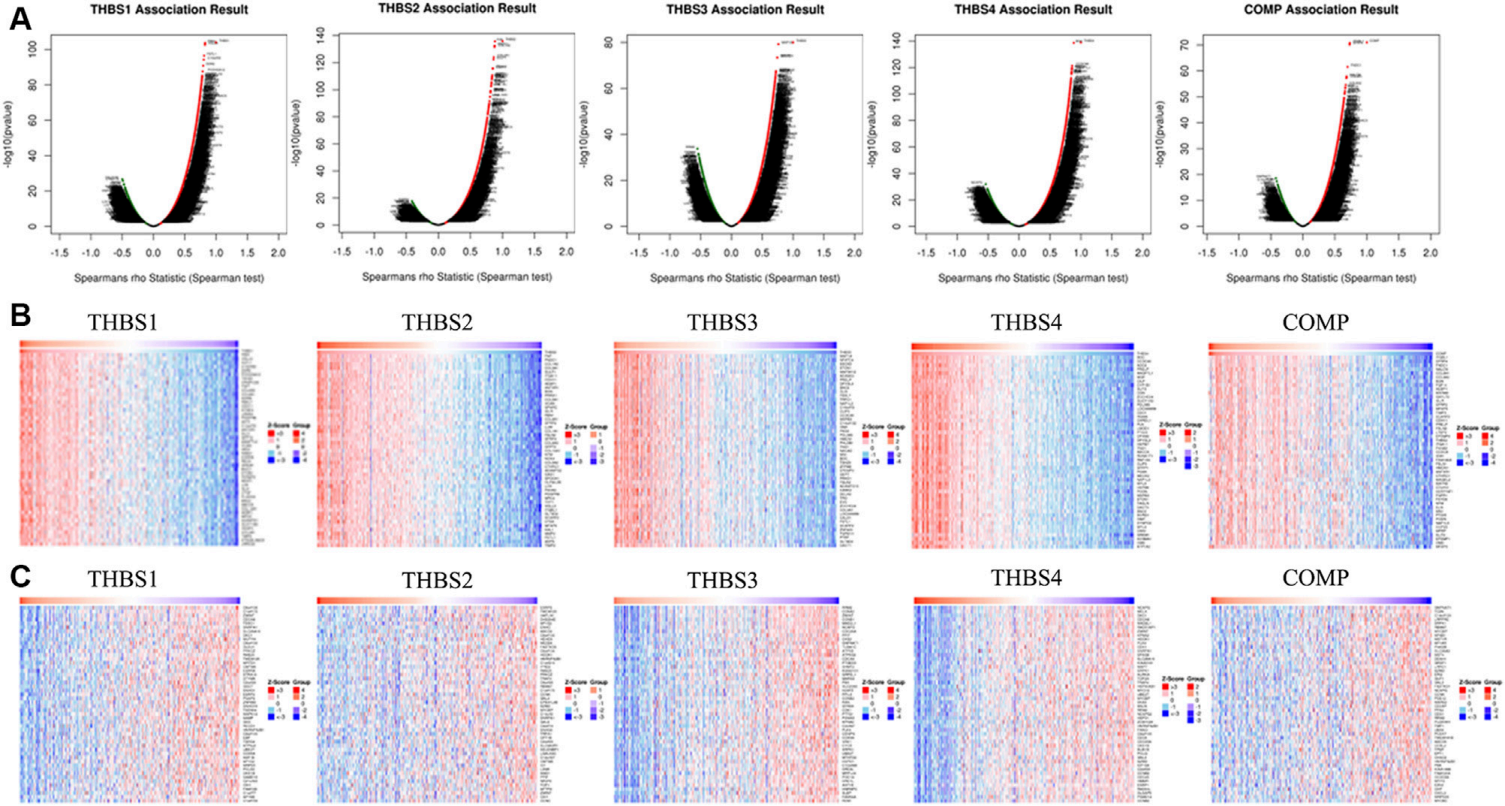
**(A)** Volcano plots showing the correlations between *THBSs* and genes differentially expressed in gastric cancer (GC); **(B)** heat maps showing top 50 genes positively correlated with *THBSs* in GC; **(C)** heat maps showing top 50 genes negatively correlated with *THBSs* in GC. Red indicates positively correlated genes and blue/green indicates negatively correlated genes (LinkedOmics).

*THBS1* expression showed a strong positive association with expression of *FBN1*, *VGLL3*, and *FSTL1*, and a strong negative correlation with *C6orf136*, *C1orf172*, and *ZWINT*. *THBS2* expression showed a strong positive association with expression of *FAP*, *FNDC1*, and *COL1A2* and a strong negative correlation with *ESRP2*, *TMEM125*, and *HAPLN*. *THBS3* expression showed a strong positive association with expression of *MAP1A*, *NFATC4*, and *SSC5D* and a strong negative correlation with *RRM2*, *CCNA2*, and *ZWINT*. *THBS4* expression showed a strong positive association with expression of *BOC*, *CCDC8*, and *AOC3* and a strong negative correlation with *NCAPG*, *MELK*, and *DKC1*. *COMP* expression showed a strong positive association with expression of *ITGBL1*, *SFRP4*, and *FNDC13* and a strong negative correlation with *GNPNAT1*, *TC2N*, and *C14orf129*. The correlations between *THBSs* and genes differentially expressed in GC are presented as volcano plots (Figure 4A), and the top 50 genes positively (Figure 4B) or negatively (Figure 4C) correlated with *THBSs* in GC are shown as heat maps. The details of the top 100 correlated genes are shown in the Supplementary Table S1.

### GO and KEGG Pathway Analysis

GO analysis presented that the genes correlated with *THBS1* were enriched in cellular response to vascular endothelial growth factor stimulus, collagen trimer, and extracellular matrix binding; for those correlated with *THBS2,* they were enriched in extracellular structure organization, ECM, and ECM binding; for those correlated with *THBS3*, they were enriched in extracellular structure organization, sarcolemma, and growth factor binding; for those correlated with *THBS4*, they were enriched in dopamine receptor signaling pathway, sarcolemma, and coreceptor activity; and for those correlated with *COMP*, they were enriched in osteoblast proliferation, endoplasmic reticulum lumen, and collagen binding. KEGG pathway analysis suggested that the genes correlated with *THBS1-5* were enriched in focal adhesion, glycosaminoglycan biosynthesis, ECM-receptor interaction, hedgehog signaling pathway, and ECM-receptor interaction, respectively (Supplementary Figure S1).

### Protein–Protein Interaction Network Analysis

**FIGURE 5:**
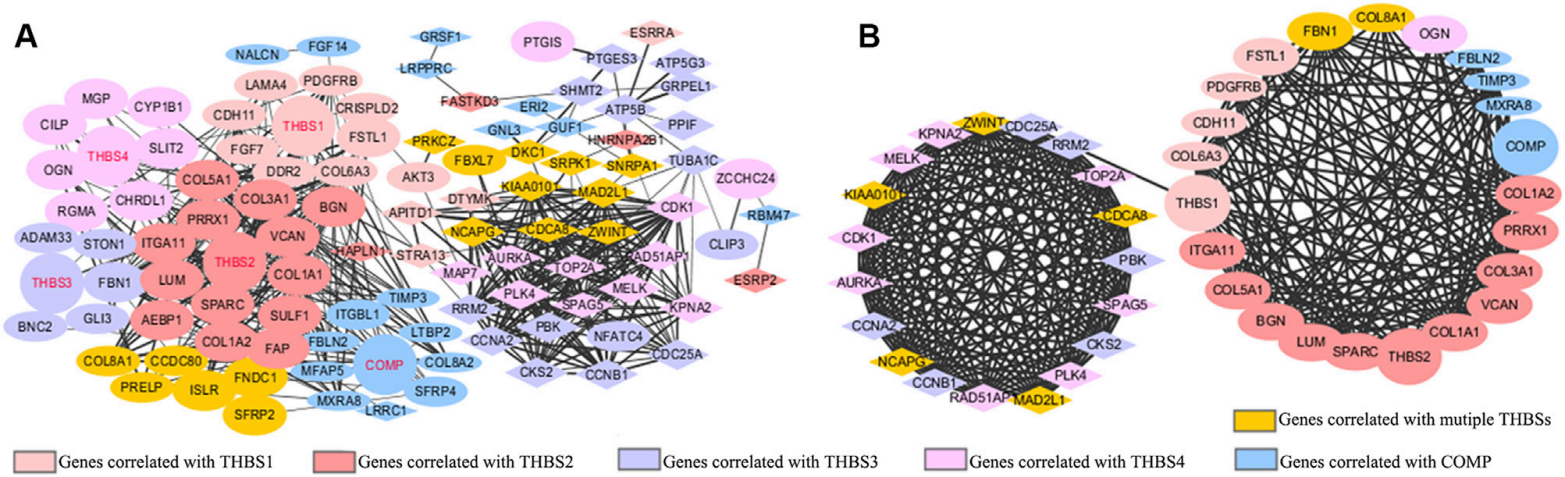
Protein–protein interaction (PPI) network of **(A)** the top 20 positively and top 20 negatively correlated genes of each *THBSs*; **(B)** potential hub genes (node degree ≥10). Ellipses stand for positively correlated genes, triangles stand for negatively correlated genes, a larger size of the node stands for a larger absolute value of Spearman correlation coefficient, and a wider line stands for a larger combined score (LinkedOmics and Cytoscape).

The PPI network mapped 106 nodes and 512 edges of the correlated genes (Figure 5A). Forty-two genes with node degree ≥10 were regarded as potential hub genes (Figure 5B).

### *THBSs* Alterations and Correlations in Gastric Cancer

**FIGURE 6:**
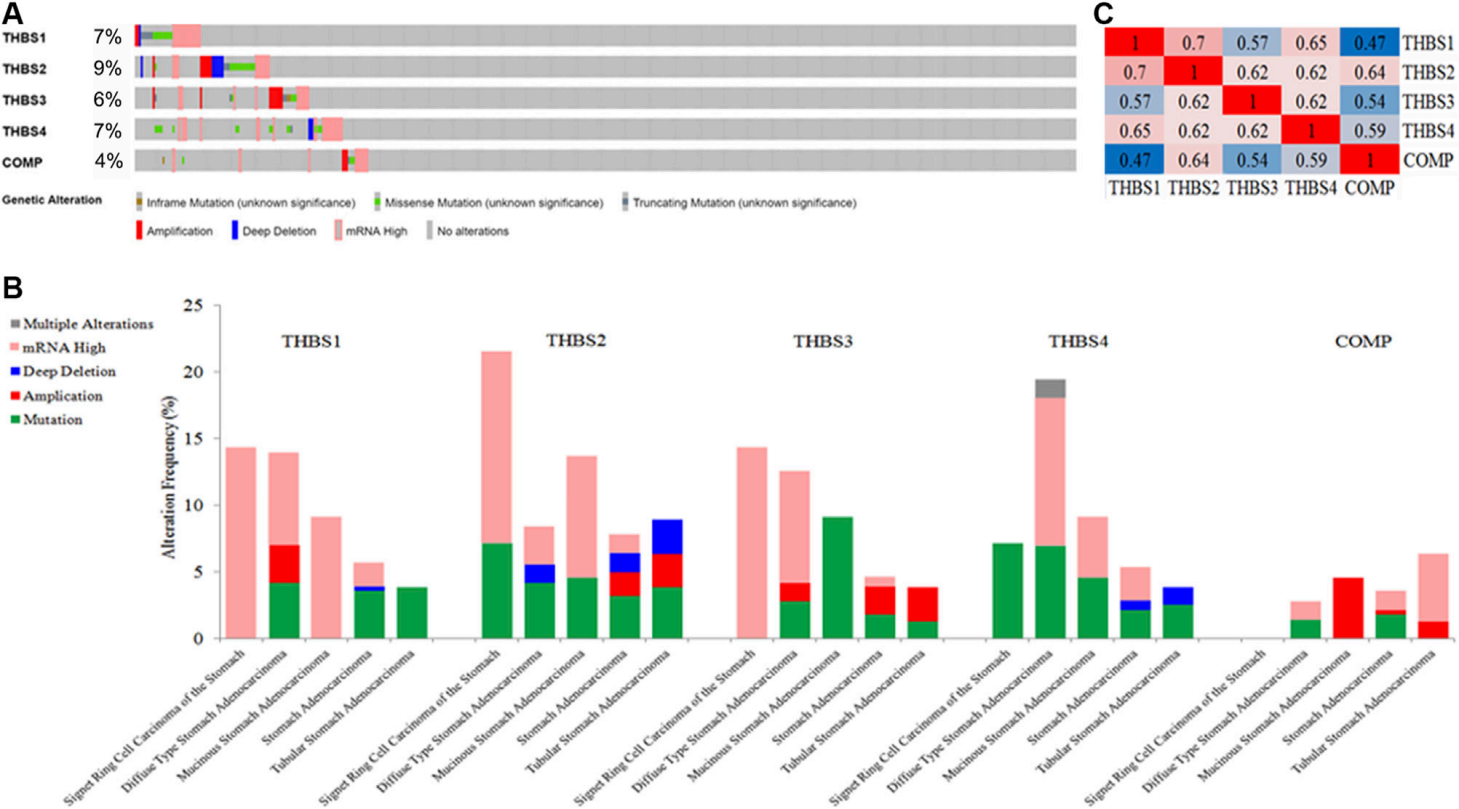
**(A)** Oncoprint of *THBSs* alterations in gastric cancer (GC); **(B)** cancer type summary of *THBSs* alterations in gastric cancer (GC); **(C)** mutual correlations of THBSs in GC (cBioportal).

*THBSs* were altered in 118 samples out of 478 (24.69%) in GC, the exact alterations of each *THBS* is presented in Figure 6A, and the alterations varied in different cancer types (Figure 6B). Mutual correlations between *THBSs* in GC are shown in heat maps in Figure 6C.

### Tumor Immunology Analysis

**FIGURE 7:**
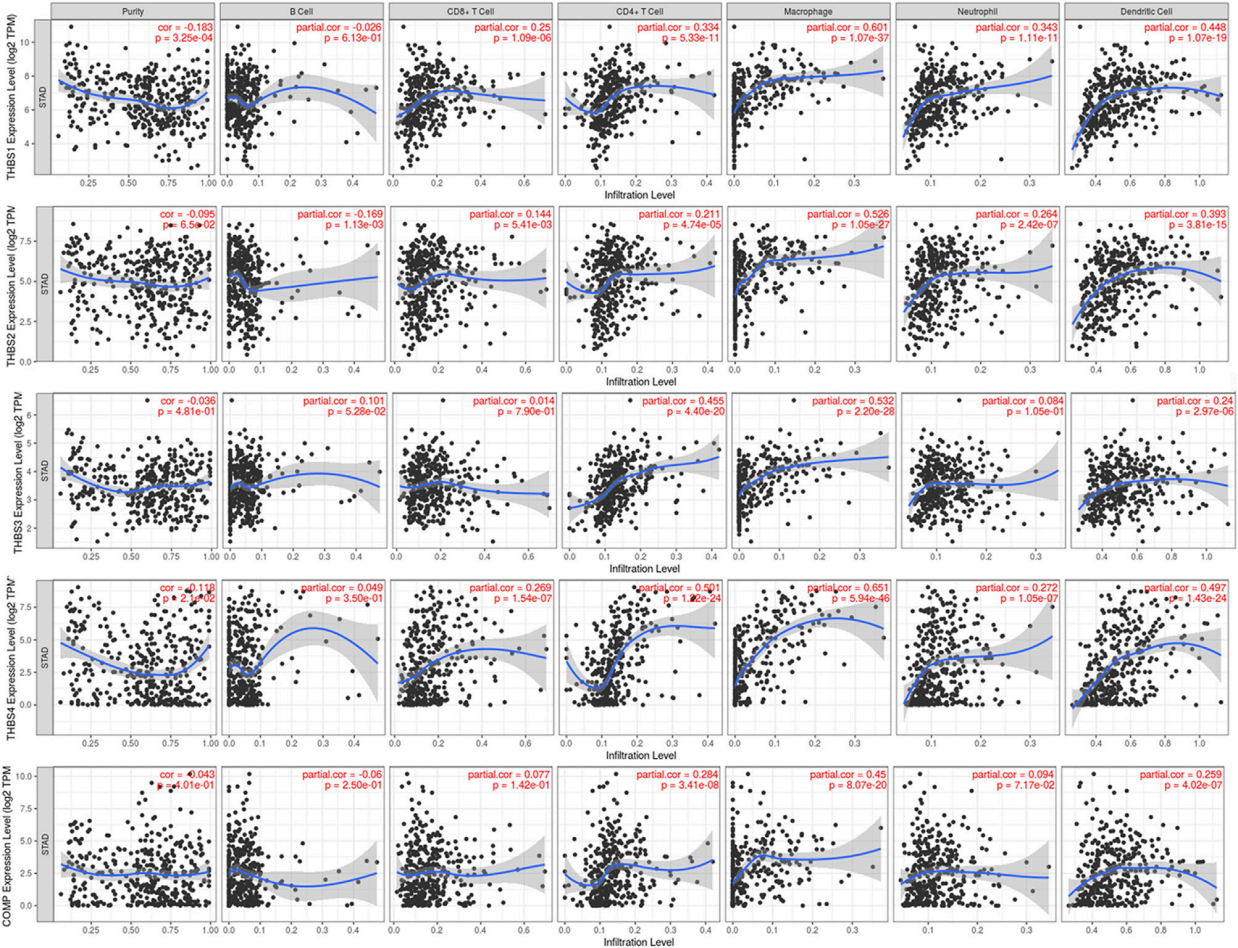
Correlations between *THBSs* expression and tumor purity and dominant immune cells infiltration levels (TIMER).

**FIGURE 8:**
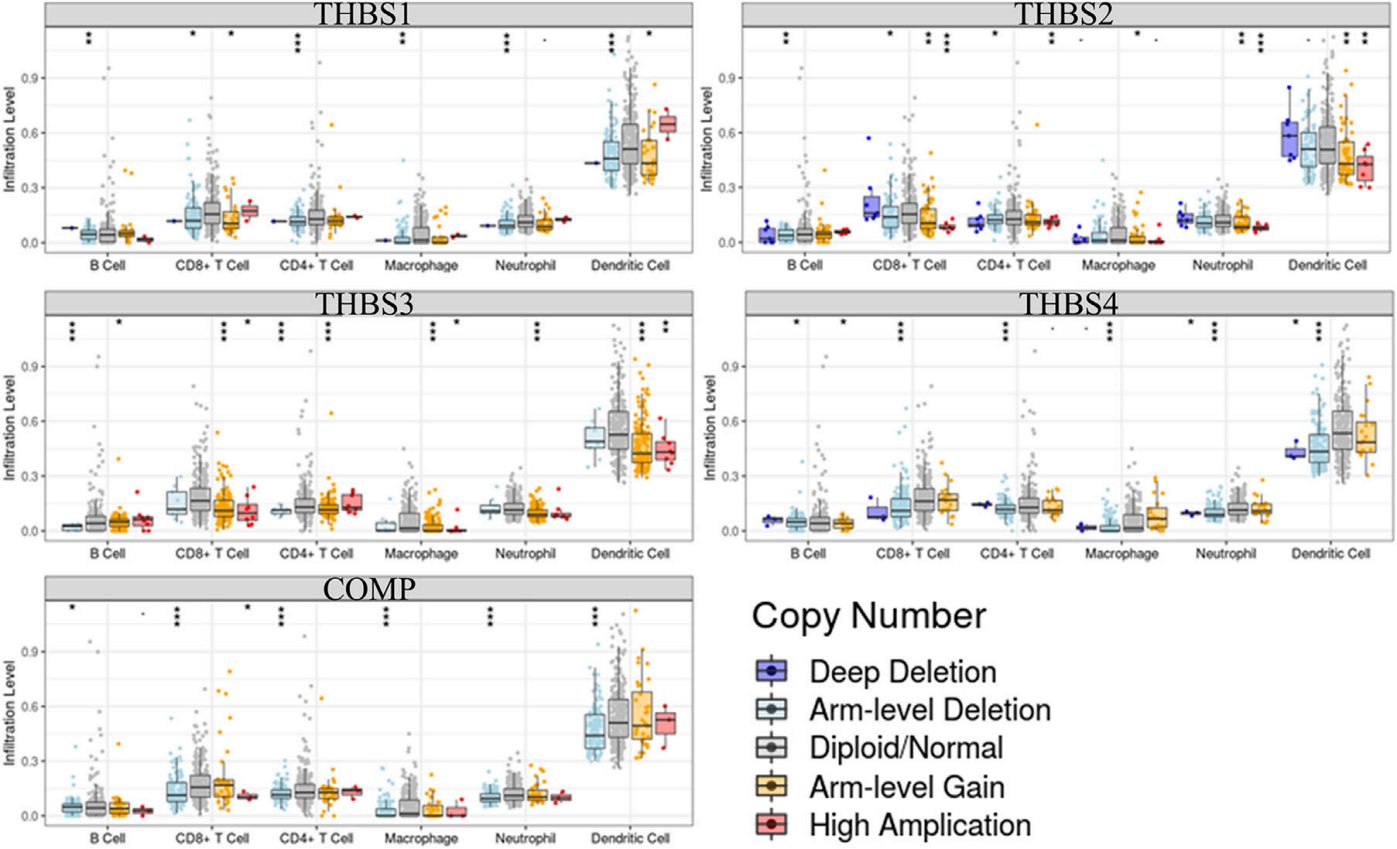
Correlations between *THBSs* somatic copy number alterations and dominant immune cells infiltration levels (significant codes: 0≤***<0.001≤**≤0.01≤*˂0.05≤●<0.1, TIMER).

Tumor immunology analysis showed that *THBS1* expression had significant correlations with tumor purity (r = −0.18, *p* = 3.3E-4) and dominant immune cells’ infiltration levels (except for B cell); *THBS2* expression had no significant correlation with tumor purity (r = −0.10, *p* = 0.07) but had significant correlations with dominant immune cells infiltration levels; *THBS3* expression had no significant correlation with tumor purity (r = −0.04, *p* = 0.48) but had significant correlations with some immune cells infiltration levels (CD4^+^ T cell, macrophage, and dendritic cell); *THBS4* expression had significant correlations with tumor purity (r = 0.12, *p* = 0.02) and dominant immune cells’ infiltration levels (except for B cell); *COMP* expression had no significant correlation with tumor purity (r = 0.04, *p* = 0.40) but had significant correlations with some immune cells infiltration levels (CD4^+^ T cell, macrophage, and dendritic cell) (Figure 7). Furthermore, *THBSs* CNA has significant correlations with dominant immune cells infiltration levels (Figure 8). Survival Kaplan–Meier in TIMER (split percentage set as 25%) showed that infiltration levels of B cell, CD8^+^ T cell, CD4^+^ T cell, neutrophil cell, and dendritic cell had no significant difference in the OS, while higher levels of macrophage associated with poor OS. Additionally, patients with high expression of *THBS1*, *THBS2*, *THBS4*, and *COMP* all had poor OS (Supplementary Figure S2). Cox proportional hazard model analysis in TIMER showed that stage 3, stage 4, age, B cell, and macrophage infiltration were risk factors for poor OS (Supplementary Table S2).

**FIGURE 9:**
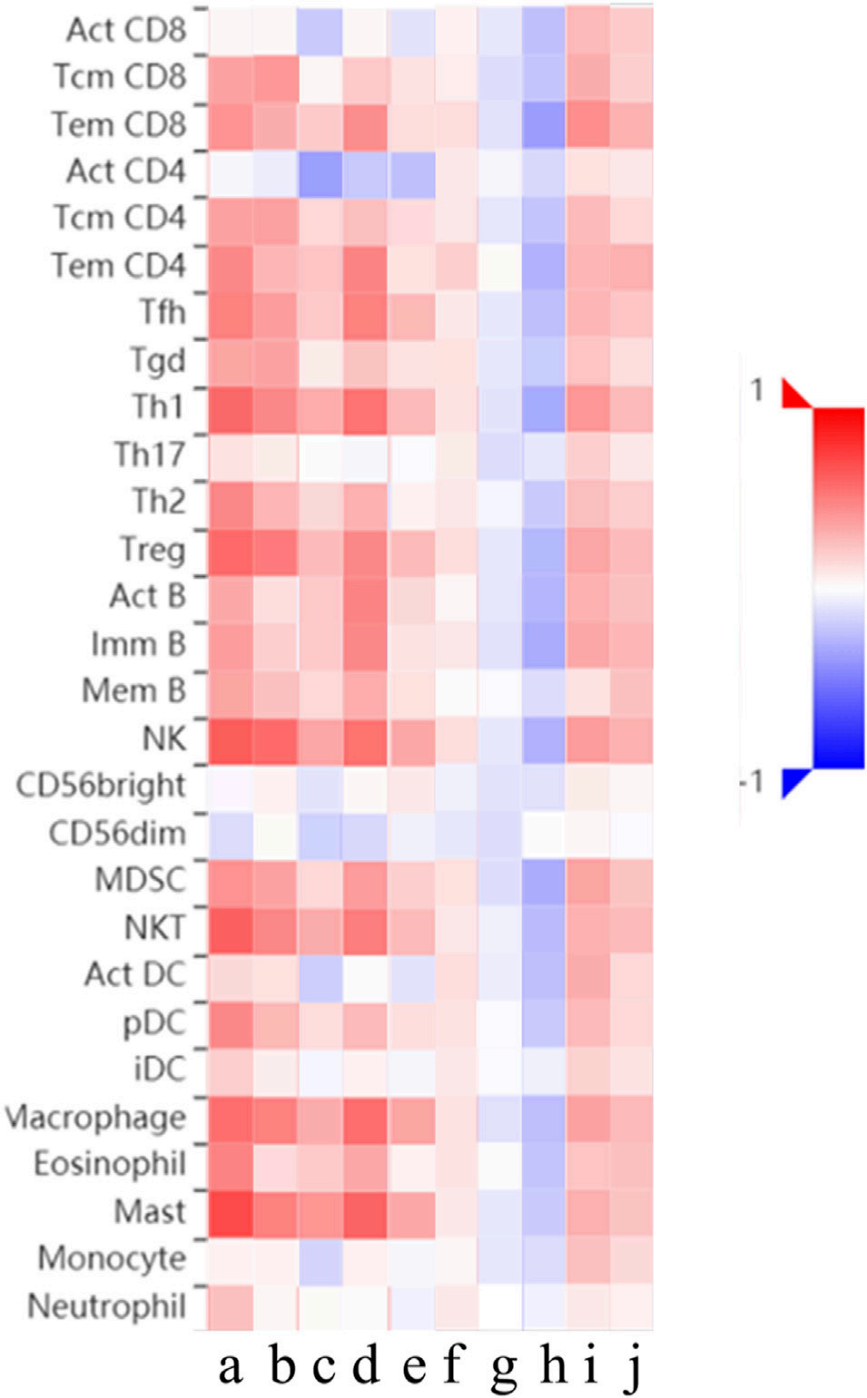
Relations between abundance of tumor-infiltrating lymphocytes and expression or copy number of *THBSs*, a–e: expression of *THBS1-5*, respectively; f–j: copy number of *THBS1-5*, respectively (TISIDB).

**FIGURE 10:**
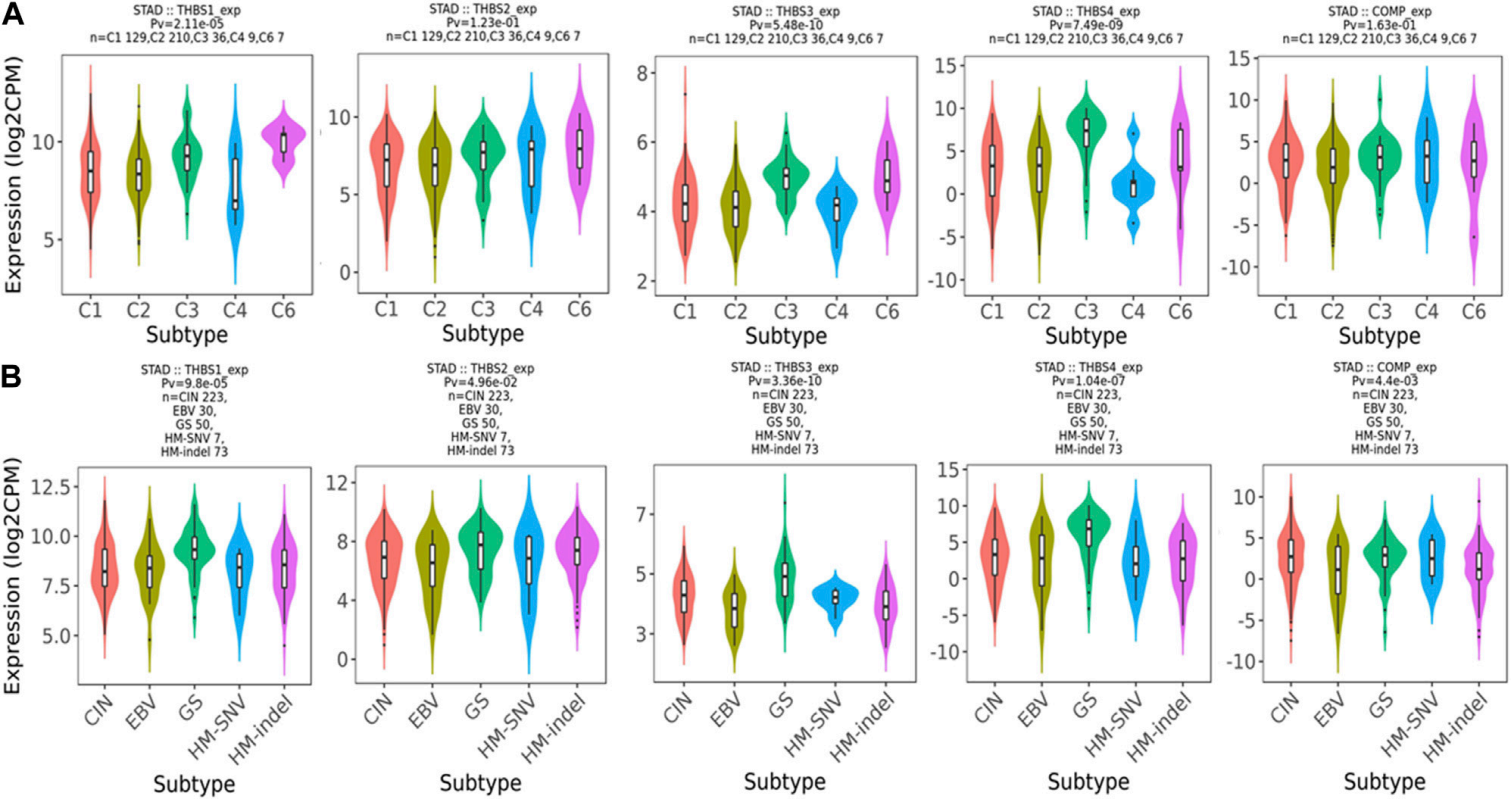
**(A)** Associations between *THBSs* expression and immune subtypes in gastric cancer (C1: wound healing; C2: IFN-γ dominant; C3: inflammatory; C4: lymphocyte depleted; C5: immunologically quiet; C6: TGF-β dominant); **(B)** associations between *THBSs* expression and molecular subtypes in gastric cancer (CIN: chromosomal instable; EBV: Epstein–Barr virus–positive; GS: genomically stable; HM-SNV: hypermutated with elevated single nucleotide variation; HM-indel: hypermutated enriched for insertion/deletion) (TISIDB).

Relations between abundance of tumor-infiltrating lymphocytes and expression or copy number of the *THBSs* are presented as heat maps in Figure 9. TISIDB further pointed out that the expression of *THBSs* had different immune subtypes in GC (Figure 10A) and that the expression of *THBSs* varied in different molecular subtypes (Figure 10B).

## Discussion

In this study, we used versatile public databases to reveal the dysregulated expression of the THBS family and their relations with tumor stage, prognosis, and tumor immunity. We mainly found that the mRNA expression levels of *THBS2*, *THBS4,* and *COMP* were significantly higher in the tumor tissues, while the expression levels of *THBS1* and *THBS3* were distinct in different databases; the expression levels of *THBS1*, *THBS2*, and *THBS4* were higher in stages 2–4 than that of stage 1; patients with high expression of *THBS1*, *THBS2*, *THBS4*, and *COMP* all had poor OS; the genes correlated with *THBSs* were enriched in focal adhesion, glycosaminoglycan biosynthesis, ECM-receptor interaction, and hedgehog signaling pathway; *THBS1* and *THBS4* expression had significant correlations with tumor purity and that all the *THBSs* expression correlated with dominant immune cells’ infiltration more or less.

THBS1 is a multifunctional matricellular glycoprotein (Guo et al., 2010), some studies showed that the mRNA levels of *THBS1* were higher in the tumor tissues than adjacent normal tissues (Lin et al., 2012; Huang et al., 2017), while this was opposite in gastric cardia adenocarcinoma (Guo et al., 2010). In our study, data from Oncomine supported that the mRNA levels of *THBS1* were higher in the tumor tissues, while data from GEPIA and UALCAN did not agree with it. From the subgroup analysis, we deduced that the inconsistency might lie in the different histopathologic types and tumor stages included in the samples. Lin et al. further proved that the mRNA levels of *THBS1* were higher in patients with larger tumors or nodal metastasis (Lin et al., 2012), which is in accordance with our results. Eto et al. selected 65 GC patients with recurrence after surgery, and they found that patients with THBS1 positive had better OS (Eto et al., 2015). In our study, we first used Kaplan–Meier plotter to perform the survival analysis, and we found that THBS1 had five probe IDs, only the probe IDs of 201110_s_at had no association with OS, whose FDR was 100%, while the other probe IDs all showed associations with OS, and results from TIMER also supported that high expression of *THBS1* had poor OS. Our result is different from theirs, the reason may lie in: 1) difference in group dividing as we set the split cutoff of low and high expression in auto select best cutoff model, and Eto et al. divided the patients as THBS1 immunohistochemistry positive or negative; 2) the number of patients included might have influence in the results. The mechanism for THBS1 associating with an aggressive tumor phenotype may happen through upregulation of matrix metalloproteinase 9, which is a key protease in cancer cell invasion and metastasis by degrading the ECM and basement membranes (Albo et al., 2002). On the other hand, tumors with strong THBS1 expression were proved to have significantly higher microvessel counts (Zhang et al., 2003).

Several studies have shown that the mRNA expression levels of *THBS2* were elevated in the tumor tissues and that higher expression correlated with later tumor stages and poorer OS (Yang et al., 2007; Zhuo et al., 2016; Ao et al., 2018), while only one study showed a positive result (Sun et al., 2014). Zhuo et al. explained that this may be due to the sample size in the latter being smaller (Zhuo et al., 2016). Our results were in accordance with the former ones. THBS2 is a matricellular Ca^2+^-binding glycoprotein excreted by stromal fibroblasts, immune cells, and endothelial cells. It plays a vital role in ECM-receptor interaction and mediating cell-to-cell and cell-to-matrix interactions (Bornstein et al., 2000). Some studies have shown that THBS2 acted as a new fibroblast growth factor-2 (FGF2) ligand that blocked FGF2 interaction with proangiogenic receptors, presenting antiangiogenic and antineoplastic activity (Rusnati et al., 2019). The conclusion may contradict with ours, the reason which probably lies in that first, THBS2 may possess multifunctional and complicated mechanisms, and other potentials have not yet been identified, and secondly, it might act differently in different cancer. As in colorectal cancer, a meta-analysis showed that high *THBS2* expression levels were correlated more often with lymph node and distant metastasis, and high levels of THBS2 expression associated with poor survival (Wang et al., 2016). While in bladder cancer, the expression levels of *THBS2* were negatively associated with tumor stages, metastasis, and grades (Nakamura et al., 2019).

Until now, we could not find any studies about THBS3 in GC. In our study, results from Oncomine indicated that the mRNA levels of *THBS3* were higher in the tumor tissues, while there was no difference in GEPIA. No association was found between the expression of *THBS3* and tumor stages, and data in Kaplan–Meier Plotter supported high expression of *THBS3* had poor OS, while data from TIMER did not. In osteosarcoma, *THBS3* was differentially expressed, and especially in patients with metastasis at diagnosis; moreover, patients with overexpressed THBS3 had worse relapse-free survival after chemotherapy (Dalla-Torre et al., 2006). In a cross-cancer genome-wide analysis, the expression quantitative trait loci results showed an association between THBS3 expression and lung cancer (Fehringer et al., 2016).

THBS4 is another extracellular secreted glycoproteins regulating the organization, repair, and remodeling of ECM. *In vitro* study showed that *THBS4* mRNA and protein levels were higher in MGC-803 and BGC-823 cells compared to normal gastric cells, and THBS4 overexpression enhanced the migration and invasion of GC cells (Chen et al., 2019). THBS4 was also reported to have strong correlations with histological type, as it was extensively overexpressed in the diffuse type, and generally lacked in intestinal type. Furthermore, immunohistochemistry demonstrated that its intensities were highest in regions with large tumor cell density and invasion (Förster et al., 2011). In our study, the mRNA levels were significantly higher in the tumor tissues and had association with tumor stages. Heat maps in Oncomine presented that the color in the diffuse type was darker than the intestinal type, in accordance with the previous study (Förster et al., 2011). Kuroda et al. showed that it was cancer-associated fibroblasts (CAFs) rather than normal-associated fibroblasts that expressed THBS4, and high expression of THBS4 was correlated with larger tumor size, more aggressiveness, lymph node metastasis, and poor OS, which was similar to our findings (Kuroda et al., 2019). Research studying the mechanism of THBS4 in GC is rare, and in endothelial cells, TGF-β1 can upregulate the THBS4 expression and affect angiogenesis, contributing to tumor growth (Muppala et al., 2017).

COMP is a soluble glycoprotein expressed in cartilage. Zhou et al. developed a gene signature consisting of COMP and five other genes, which correlated with recurrence of patients with GC in stages III and IV (Zhou et al., 2018). Another study also proved that COMP hypomethylation was associated with poor OS (Liang et al., 2019). These results were similar to ours. The regulatory mechanisms of COMP in GC are unknown. Papadakos et al. proved that breast cancer cells expressing COMP formed larger size tumors *in vivo* and *in vitro* and that COMP could activate Notch3, interacting with both Notch3 and its ligand Jagged1, and they may also interact with β-catenin and AKT pathways (Papadakos et al., 2019).

## Conclusion

Our results implied that THBS2, THBS4, and COMP were potentially diagnostic markers for GC; THBS1, THBS2, THBS4, and COMP were potentially prognostic markers for GC; the function and regulatory mechanisms of THBSs in GC might happen through focal adhesion, glycosaminoglycan biosynthesis, ECM-receptor interaction, and hedgehog signaling pathway; investigating the relations of THBSs and tumor immunology might help in immunotherapy in GC. The results were based on multidimensional bioinformatic analysis, and several databases have been used to verify the results with each other, but a small part of the results were not consistent with the previously published ones; hence, more studies are still needed to confirm these results.

## Data Availability

The original contributions presented in the study are included in the article/Supplementary Material, further inquiries can be directed to the corresponding author.
