# Supplementary material for: Diagnostic, Therapeutic, and Prognostic Value of the Thrombospondin Family in Gastric Cancer
Source: Front Mol Biosci. 2021 Apr 28;8:647095. doi: 10.3389/fmolb.2021.647095 (PMC8113821; doi:10.3389/fmolb.2021.647095)
Supplement: Supplementary file 1 [file Table1.docx]

Table S1 Top 100 genes positively or negatively correlated with *THBSs* in gastric cancer (LinkedOmics)

| GENE | Query | Statistic | P-value | FDR (BH) |  | Query | Statistic | P-value | FDR (BH) |
| --- | --- | --- | --- | --- | --- | --- | --- | --- | --- |
| *THBS1* | *FBN1* | 0.83 | 1.79E-104 | 1.81E-100 |  | *C6orf136* | -0.50 | 2.14E-27 | 3.69E-26 |
|  | *VGLL3* | 0.82 | 2.27E-103 | 1.53E-99 |  | *KDF1* | -0.49 | 2.20E-26 | 3.56E-25 |
|  | *FSTL1* | 0.81 | 3.64E-97 | 1.84E-93 |  | *ZWINT* | -0.47 | 1.35E-24 | 1.95E-23 |
|  | *MEDAG* | 0.80 | 6.18E-95 | 2.50E-91 |  | *CDCA8* | -0.47 | 3.20E-24 | 4.50E-23 |
|  | *DDR2* | 0.79 | 1.42E-91 | 4.79E-88 |  | *TSSC1* | -0.46 | 8.76E-23 | 1.15E-21 |
|  | *PCDHGA12* | 0.79 | 3.17E-88 | 9.17E-85 |  | *SNRPA1* | -0.45 | 1.49E-22 | 1.93E-21 |
|  | *TSHZ3* | 0.78 | 6.48E-86 | 1.64E-82 |  | *SLC25A10* | -0.45 | 3.22E-22 | 4.08E-21 |
|  | *CRISPLD2* | 0.78 | 8.29E-86 | 1.86E-82 |  | *DKC1* | -0.45 | 3.34E-22 | 4.22E-21 |
|  | *FGF7* | 0.78 | 2.03E-85 | 4.11E-82 |  | *MUTYH* | -0.45 | 5.20E-22 | 6.54E-21 |
|  | *COL6A3* | 0.78 | 6.04E-85 | 1.11E-81 |  | *OARD1* | -0.45 | 6.51E-22 | 8.13E-21 |
|  | *COL8A1* | 0.78 | 1.09E-84 | 1.73E-81 |  | *DLEU1* | -0.45 | 6.89E-22 | 8.57E-21 |
|  | *ASAM* | 0.78 | 1.11E-84 | 1.73E-81 |  | *PRKCZ* | -0.45 | 1.01E-21 | 1.25E-20 |
|  | *FBXL7* | 0.78 | 1.61E-84 | 2.33E-81 |  | *RAB25* | -0.45 | 1.18E-21 | 1.45E-20 |
|  | *CDH11* | 0.77 | 5.92E-84 | 7.98E-81 |  | *TMEM125* | -0.44 | 1.61E-21 | 1.96E-20 |
|  | *KCNE4* | 0.77 | 2.65E-83 | 3.36E-80 |  | *APITD1* | -0.44 | 3.90E-21 | 4.65E-20 |
|  | *LAMA4* | 0.77 | 5.37E-83 | 6.38E-80 |  | *CMTM8* | -0.44 | 3.94E-21 | 4.70E-20 |
|  | *PDGFRB* | 0.77 | 1.16E-82 | 1.31E-79 |  | *ESRRA* | -0.44 | 4.61E-21 | 5.48E-20 |
|  | *AKT3* | 0.77 | 2.17E-82 | 2.31E-79 |  | *STRA13* | -0.44 | 5.83E-21 | 6.89E-20 |
|  | *VSTM4* | 0.77 | 2.81E-81 | 2.84E-78 |  | *DTYMK* | -0.44 | 6.31E-21 | 7.43E-20 |
|  | *ANTXR1* | 0.77 | 6.11E-81 | 5.88E-78 |  | *CNOT11* | -0.44 | 8.38E-21 | 9.79E-20 |
|  | *DLC1* | 0.76 | 7.64E-81 | 7.02E-78 |  | *GGCT* | -0.44 | 1.07E-20 | 1.24E-19 |
|  | *GFPT2* | 0.76 | 5.45E-80 | 4.79E-77 |  | *SNHG1* | -0.44 | 1.16E-20 | 1.35E-19 |
|  | *ANGPTL2* | 0.76 | 2.46E-79 | 2.07E-76 |  | *ESRP2* | -0.44 | 1.30E-20 | 1.51E-19 |
|  | *VCAN* | 0.76 | 3.89E-79 | 3.14E-76 |  | *PGAP2* | -0.44 | 1.32E-20 | 1.53E-19 |
|  | *HEG1* | 0.76 | 9.58E-79 | 7.45E-76 |  | *ZNF692* | -0.43 | 1.60E-20 | 1.84E-19 |
|  | *RAB31* | 0.76 | 1.58E-78 | 1.18E-75 |  | *SNHG10* | -0.43 | 1.62E-20 | 1.86E-19 |
|  | *EDNRA* | 0.76 | 2.58E-78 | 1.86E-75 |  | *TSEN54* | -0.43 | 1.66E-20 | 1.91E-19 |
|  | *RECK* | 0.76 | 3.22E-78 | 2.24E-75 |  | *MAPK13* | -0.43 | 2.51E-20 | 2.86E-19 |
|  | *GREM1* | 0.76 | 6.93E-78 | 4.67E-75 |  | *AAMP* | -0.43 | 3.10E-20 | 3.52E-19 |
|  | *BICC1* | 0.75 | 1.31E-77 | 8.52E-75 |  | *GSS* | -0.43 | 3.96E-20 | 4.46E-19 |
|  | *STON1* | 0.75 | 1.53E-77 | 9.66E-75 |  | *RCCD1* | -0.43 | 6.14E-20 | 6.81E-19 |
|  | *FERMT2* | 0.75 | 3.14E-77 | 1.92E-74 |  | *HNRNPA2B1* | -0.43 | 7.55E-20 | 8.34E-19 |
|  | *WISP1* | 0.75 | 4.12E-77 | 2.45E-74 |  | *ARHGEF39* | -0.43 | 9.76E-20 | 1.07E-18 |
|  | *LOX* | 0.75 | 7.72E-77 | 4.46E-74 |  | *EBP* | -0.42 | 1.29E-19 | 1.40E-18 |
|  | *GLI3* | 0.75 | 1.09E-76 | 6.10E-74 |  | *TBRG4* | -0.42 | 1.46E-19 | 1.58E-18 |
|  | *CTGF* | 0.75 | 1.25E-76 | 6.85E-74 |  | *ATP5J2* | -0.42 | 1.57E-19 | 1.70E-18 |
|  | *PLXDC2* | 0.75 | 3.83E-76 | 2.04E-73 |  | *UBE2T* | -0.42 | 2.62E-19 | 2.80E-18 |
|  | *BNC2* | 0.75 | 5.40E-76 | 2.80E-73 |  | *COX5A* | -0.42 | 2.87E-19 | 3.05E-18 |
|  | *ABCC9* | 0.75 | 6.67E-76 | 3.37E-73 |  | *ASF1B* | -0.42 | 3.00E-19 | 3.19E-18 |
|  | *COL14A1* | 0.75 | 4.47E-75 | 2.21E-72 |  | *AP1G2* | -0.42 | 3.04E-19 | 3.23E-18 |
|  | *AEBP1* | 0.74 | 1.60E-74 | 7.71E-72 |  | *MRPS5* | -0.42 | 3.38E-19 | 3.57E-18 |
|  | *MPDZ* | 0.74 | 1.64E-74 | 7.71E-72 |  | *POLD2* | -0.42 | 3.38E-19 | 3.57E-18 |
|  | *ADAMTS1* | 0.74 | 3.01E-74 | 1.38E-71 |  | *CKS1B* | -0.42 | 4.24E-19 | 4.45E-18 |
|  | *GUCY1B3* | 0.74 | 6.38E-74 | 2.87E-71 |  | *SAMD10* | -0.42 | 4.77E-19 | 4.99E-18 |
|  | *VEGFC* | 0.74 | 1.37E-73 | 6.01E-71 |  | *MIS18A* | -0.42 | 7.38E-19 | 7.65E-18 |
|  | *COL3A1* | 0.74 | 1.41E-73 | 6.08E-71 |  | *IDH1* | -0.42 | 9.48E-19 | 9.80E-18 |
|  | *TIMP2* | 0.74 | 1.52E-73 | 6.41E-71 |  | *FAM72B* | -0.42 | 9.97E-19 | 1.03E-17 |
|  | *ST6GALNAC5* | 0.74 | 1.66E-73 | 6.85E-71 |  | *CHTOP* | -0.41 | 1.21E-18 | 1.24E-17 |
|  | *LRRC32* | 0.74 | 1.97E-73 | 7.96E-71 |  | *AP1M2* | -0.41 | 1.43E-18 | 1.46E-17 |
|  | *LUM* | 0.74 | 3.15E-73 | 1.25E-70 |  | *C1orf159* | -0.41 | 1.49E-18 | 1.52E-17 |
|  | *IL1R1* | 0.74 | 5.89E-73 | 2.29E-70 |  | *RAB17* | -0.41 | 1.52E-18 | 1.55E-17 |
|  | *FNDC1* | 0.74 | 2.60E-72 | 9.92E-70 |  | *UBE2C* | -0.41 | 1.61E-18 | 1.63E-17 |
|  | *GLT8D2* | 0.74 | 4.00E-72 | 1.50E-69 |  | *PPIH* | -0.41 | 1.71E-18 | 1.73E-17 |
|  | *GPR124* | 0.74 | 4.69E-72 | 1.72E-69 |  | *TRAF4* | -0.41 | 1.77E-18 | 1.79E-17 |
|  | *TMEM47* | 0.74 | 5.55E-72 | 2.00E-69 |  | *SLC5A6* | -0.41 | 1.89E-18 | 1.90E-17 |
|  | *KIRREL* | 0.74 | 8.75E-72 | 3.11E-69 |  | *MAP7* | -0.41 | 2.59E-18 | 2.59E-17 |
|  | *SVEP1* | 0.74 | 1.10E-71 | 3.83E-69 |  | *ATIC* | -0.41 | 2.73E-18 | 2.72E-17 |
|  | *ZNF521* | 0.73 | 1.09E-70 | 3.74E-68 |  | *ZFAND2B* | -0.41 | 2.97E-18 | 2.95E-17 |
|  | *MMP2* | 0.73 | 1.30E-70 | 4.39E-68 |  | *HJURP* | -0.41 | 3.37E-18 | 3.34E-17 |
|  | *PCDHGB7* | 0.73 | 1.63E-70 | 5.39E-68 |  | *BCS1L* | -0.41 | 3.48E-18 | 3.43E-17 |
|  | *SYDE1* | 0.73 | 7.68E-70 | 2.49E-67 |  | *LSM5* | -0.41 | 4.01E-18 | 3.95E-17 |
|  | *NRP2* | 0.73 | 7.74E-70 | 2.49E-67 |  | *ACP1* | -0.41 | 4.35E-18 | 4.27E-17 |
|  | *LAMA2* | 0.73 | 8.88E-70 | 2.81E-67 |  | *RECQL4* | -0.41 | 4.50E-18 | 4.41E-17 |
|  | *NID2* | 0.73 | 1.10E-69 | 3.43E-67 |  | *HDGF* | -0.41 | 5.36E-18 | 5.22E-17 |
|  | *PRRX1* | 0.73 | 2.27E-69 | 6.97E-67 |  | *FASTKD3* | -0.41 | 5.81E-18 | 5.65E-17 |
|  | *ARHGAP20* | 0.73 | 3.12E-69 | 9.35E-67 |  | *ELMO3* | -0.41 | 5.94E-18 | 5.77E-17 |
|  | *CCDC80* | 0.73 | 3.14E-69 | 9.35E-67 |  | *EME1* | -0.41 | 6.49E-18 | 6.28E-17 |
|  | *GALNTL2* | 0.73 | 6.69E-69 | 1.96E-66 |  | *AK2* | -0.41 | 6.70E-18 | 6.48E-17 |
|  | *ZEB2* | 0.72 | 7.64E-69 | 2.21E-66 |  | *TMEM45B* | -0.41 | 6.72E-18 | 6.49E-17 |
|  | *SULF1* | 0.72 | 1.02E-68 | 2.91E-66 |  | *DPH2* | -0.41 | 7.67E-18 | 7.37E-17 |
|  | *NHSL2* | 0.72 | 1.12E-68 | 3.16E-66 |  | *NSDHL* | -0.40 | 9.20E-18 | 8.80E-17 |
|  | *DCN* | 0.72 | 1.33E-68 | 3.68E-66 |  | *KIAA0101* | -0.40 | 9.23E-18 | 8.82E-17 |
|  | *GAS1* | 0.72 | 1.77E-68 | 4.85E-66 |  | *CDK1* | -0.40 | 9.56E-18 | 9.12E-17 |
|  | *MSRB3* | 0.72 | 2.52E-68 | 6.79E-66 |  | *EPCAM* | -0.40 | 1.28E-17 | 1.22E-16 |
|  | *PODN* | 0.72 | 1.14E-67 | 3.03E-65 |  | *IFRD2* | -0.40 | 1.43E-17 | 1.35E-16 |
|  | *PRKD1* | 0.72 | 1.75E-67 | 4.59E-65 |  | *PSMG3* | -0.40 | 1.44E-17 | 1.36E-16 |
|  | *KCNJ8* | 0.72 | 2.58E-67 | 6.69E-65 |  | *SNRPA* | -0.40 | 1.44E-17 | 1.36E-16 |
|  | *PRKG1* | 0.72 | 4.07E-67 | 1.04E-64 |  | *C1orf210* | -0.40 | 1.55E-17 | 1.46E-16 |
|  | *LAYN* | 0.72 | 4.20E-67 | 1.06E-64 |  | *HDHD3* | -0.40 | 1.59E-17 | 1.50E-16 |
|  | *FAM180A* | 0.72 | 4.85E-67 | 1.21E-64 |  | *CNKSR1* | -0.40 | 1.76E-17 | 1.65E-16 |
|  | *AOC3* | 0.72 | 5.65E-67 | 1.39E-64 |  | *FAAH* | -0.40 | 1.76E-17 | 1.65E-16 |
|  | *MAP1A* | 0.72 | 6.07E-67 | 1.48E-64 |  | *ARHGEF16* | -0.40 | 1.81E-17 | 1.69E-16 |
|  | *TIMP3* | 0.72 | 1.10E-66 | 2.65E-64 |  | *OXSM* | -0.40 | 2.11E-17 | 1.96E-16 |
|  | *CLIC4* | 0.72 | 1.68E-66 | 4.00E-64 |  | *FAM195A* | -0.40 | 2.13E-17 | 1.99E-16 |
|  | *A2M* | 0.72 | 2.34E-66 | 5.50E-64 |  | *LOC81691* | -0.40 | 2.29E-17 | 2.13E-16 |
|  | *FAM26E* | 0.71 | 5.46E-66 | 1.27E-63 |  | *WBSCR22* | -0.40 | 2.49E-17 | 2.31E-16 |
|  | *HTR2A* | 0.71 | 5.54E-66 | 1.27E-63 |  | *CDC20* | -0.40 | 2.53E-17 | 2.34E-16 |
|  | *COL5A2* | 0.71 | 6.67E-66 | 1.52E-63 |  | *C13orf34* | -0.40 | 2.54E-17 | 2.35E-16 |
|  | *PDLIM3* | 0.71 | 8.80E-66 | 1.98E-63 |  | *FRAT2* | -0.40 | 2.54E-17 | 2.35E-16 |
|  | *MFAP5* | 0.71 | 9.10E-66 | 2.02E-63 |  | *RASSF7* | -0.40 | 3.33E-17 | 3.06E-16 |
|  | *PTGER3* | 0.71 | 9.48E-66 | 2.08E-63 |  | *SFXN4* | -0.40 | 3.36E-17 | 3.09E-16 |
|  | *RASGRF2* | 0.71 | 1.25E-65 | 2.72E-63 |  | *MTX2* | -0.40 | 3.41E-17 | 3.13E-16 |
|  | *C1R* | 0.71 | 1.50E-65 | 3.23E-63 |  | *C11orf35* | -0.40 | 3.59E-17 | 3.29E-16 |
|  | *FIBIN* | 0.71 | 2.76E-65 | 5.87E-63 |  | *GRTP1* | -0.40 | 3.59E-17 | 3.29E-16 |
|  | *CHSY3* | 0.71 | 4.82E-65 | 1.02E-62 |  | *STAMBP* | -0.40 | 3.69E-17 | 3.38E-16 |
|  | *LPPR4* | 0.71 | 4.98E-65 | 1.04E-62 |  | *GINS1* | -0.40 | 3.82E-17 | 3.49E-16 |
|  | *COL6A2* | 0.71 | 7.07E-65 | 1.46E-62 |  | *NUF2* | -0.40 | 3.91E-17 | 3.56E-16 |
|  | *C1S* | 0.71 | 1.11E-64 | 2.28E-62 |  | *WHSC2* | -0.40 | 4.23E-17 | 3.85E-16 |
|  | *ATP8B2* | 0.71 | 1.43E-64 | 2.89E-62 |  | *HMBS* | -0.40 | 4.42E-17 | 4.01E-16 |
|  | *SPOCK1* | 0.71 | 1.97E-64 | 3.95E-62 |  | *TROAP* | -0.40 | 4.44E-17 | 4.02E-16 |
| *THBS2* | *FAP* | 0.88 | 1.83E-136 | 1.85E-132 |  | *ESRP2* | -0.41 | 1.93E-18 | 3.63E-17 |
|  | *FNDC1* | 0.88 | 2.23E-133 | 1.51E-129 |  | *TMEM125* | -0.40 | 3.48E-17 | 5.90E-16 |
|  | *COL1A2* | 0.88 | 2.72E-132 | 1.38E-128 |  | *HAPLN1* | -0.39 | 8.97E-17 | 1.47E-15 |
|  | *COL3A1* | 0.86 | 8.18E-125 | 3.31E-121 |  | *D4S234E* | -0.39 | 3.72E-16 | 5.79E-15 |
|  | *SULF1* | 0.86 | 3.93E-123 | 1.32E-119 |  | *AP1G2* | -0.37 | 4.52E-15 | 6.56E-14 |
|  | *ITGA11* | 0.85 | 1.21E-116 | 3.49E-113 |  | *ENHO* | -0.37 | 1.09E-14 | 1.54E-13 |
|  | *CDH11* | 0.85 | 3.39E-116 | 8.57E-113 |  | *ABCD3* | -0.36 | 2.85E-14 | 3.92E-13 |
|  | *AEBP1* | 0.84 | 1.88E-111 | 4.22E-108 |  | *OARD1* | -0.36 | 3.52E-14 | 4.79E-13 |
|  | *ANTXR1* | 0.84 | 8.75E-111 | 1.77E-107 |  | *HDHD3* | -0.36 | 4.98E-14 | 6.68E-13 |
|  | *BGN* | 0.84 | 2.10E-110 | 3.86E-107 |  | *MED24* | -0.36 | 5.04E-14 | 6.75E-13 |
|  | *PRRX1* | 0.84 | 5.27E-110 | 8.88E-107 |  | *FASTKD3* | -0.35 | 9.25E-14 | 1.21E-12 |
|  | *COL5A1* | 0.84 | 2.90E-109 | 4.51E-106 |  | *C6orf136* | -0.35 | 1.09E-13 | 1.41E-12 |
|  | *VCAN* | 0.83 | 3.80E-109 | 5.49E-106 |  | *HOOK1* | -0.35 | 1.36E-13 | 1.75E-12 |
|  | *SPARC* | 0.83 | 2.10E-108 | 2.83E-105 |  | *HNRNPA2B1* | -0.35 | 2.24E-13 | 2.82E-12 |
|  | *ISLR* | 0.83 | 2.54E-106 | 3.16E-103 |  | *C1orf210* | -0.35 | 2.39E-13 | 3.01E-12 |
|  | *FBN1* | 0.83 | 2.66E-106 | 3.16E-103 |  | *IFRD2* | -0.35 | 2.67E-13 | 3.36E-12 |
|  | *COL8A1* | 0.83 | 5.10E-106 | 5.73E-103 |  | *RAB25* | -0.35 | 4.47E-13 | 5.53E-12 |
|  | *SFRP2* | 0.83 | 7.79E-105 | 8.29E-102 |  | *PRKCZ* | -0.34 | 6.19E-13 | 7.57E-12 |
|  | *LUM* | 0.82 | 1.81E-103 | 1.83E-100 |  | *TRAF4* | -0.34 | 7.30E-13 | 8.88E-12 |
|  | *COL1A1* | 0.82 | 3.16E-103 | 3.04E-100 |  | *SAPCD1* | -0.34 | 7.52E-13 | 9.13E-12 |
|  | *FBLN2* | 0.81 | 6.50E-100 | 5.98E-97 |  | *RBM47* | -0.34 | 9.78E-13 | 1.17E-11 |
|  | *SFRP4* | 0.81 | 1.01E-99 | 8.86E-97 |  | *KDF1* | -0.34 | 1.09E-12 | 1.29E-11 |
|  | *COL6A3* | 0.81 | 2.66E-99 | 2.24E-96 |  | *CCNK* | -0.34 | 1.46E-12 | 1.71E-11 |
|  | *GFPT2* | 0.81 | 9.51E-99 | 7.69E-96 |  | *GNL3* | -0.34 | 1.51E-12 | 1.76E-11 |
|  | *COL10A1* | 0.81 | 9.91E-96 | 7.71E-93 |  | *EPB41L4B* | -0.34 | 1.55E-12 | 1.81E-11 |
|  | *NTM* | 0.80 | 2.01E-95 | 1.51E-92 |  | *BZW2* | -0.34 | 1.77E-12 | 2.05E-11 |
|  | *NOX4* | 0.80 | 2.31E-92 | 1.67E-89 |  | *MYCBP* | -0.34 | 1.78E-12 | 2.06E-11 |
|  | *COL5A2* | 0.80 | 2.73E-92 | 1.90E-89 |  | *ATP5MPL* | -0.33 | 2.88E-12 | 3.29E-11 |
|  | *CTHRC1* | 0.79 | 1.64E-90 | 1.11E-87 |  | *SNRPA1* | -0.33 | 3.09E-12 | 3.52E-11 |
|  | *ADAMTS2* | 0.79 | 6.72E-90 | 4.38E-87 |  | *GALE* | -0.33 | 4.08E-12 | 4.61E-11 |
|  | *GAS1* | 0.79 | 1.15E-89 | 7.28E-87 |  | *C4orf19* | -0.33 | 4.61E-12 | 5.18E-11 |
|  | *SPOCK1* | 0.79 | 1.61E-89 | 9.89E-87 |  | *SNHG3* | -0.33 | 6.18E-12 | 6.85E-11 |
|  | *OLFML2B* | 0.79 | 1.71E-89 | 1.01E-86 |  | *TRPA1* | -0.33 | 7.78E-12 | 8.55E-11 |
|  | *LOX* | 0.79 | 4.05E-89 | 2.34E-86 |  | *CPT1B* | -0.33 | 8.97E-12 | 9.80E-11 |
|  | *P4HA3* | 0.79 | 1.11E-88 | 6.10E-86 |  | *CNOT11* | -0.33 | 1.04E-11 | 1.14E-10 |
|  | *PDGFRB* | 0.79 | 1.12E-88 | 6.10E-86 |  | *SLC9A3R1* | -0.33 | 1.05E-11 | 1.14E-10 |
|  | *MRC2* | 0.79 | 4.65E-88 | 2.48E-85 |  | *SELENBP1* | -0.32 | 1.24E-11 | 1.34E-10 |
|  | *THY1* | 0.78 | 8.86E-88 | 4.59E-85 |  | *LGALS9C* | -0.32 | 1.25E-11 | 1.35E-10 |
|  | *VGLL3* | 0.78 | 2.13E-87 | 1.08E-84 |  | *HNF1A-AS1* | -0.32 | 1.28E-11 | 1.38E-10 |
|  | *ITGBL1* | 0.78 | 3.21E-86 | 1.59E-83 |  | *CMTM8* | -0.32 | 1.45E-11 | 1.55E-10 |
|  | *GLT8D2* | 0.78 | 6.61E-85 | 3.18E-82 |  | *ID1* | -0.32 | 1.46E-11 | 1.57E-10 |
|  | *SCARF2* | 0.77 | 5.34E-84 | 2.51E-81 |  | *LSM5* | -0.32 | 1.71E-11 | 1.81E-10 |
|  | *CTSK* | 0.77 | 1.02E-82 | 4.69E-80 |  | *AMD1* | -0.32 | 1.71E-11 | 1.82E-10 |
|  | *MFAP5* | 0.77 | 1.43E-82 | 6.41E-80 |  | *PPIF* | -0.32 | 1.78E-11 | 1.88E-10 |
|  | *KAL1* | 0.76 | 2.44E-80 | 1.06E-77 |  | *NR2F6* | -0.32 | 1.87E-11 | 1.97E-10 |
|  | *MMP2* | 0.76 | 2.47E-80 | 1.06E-77 |  | *TOP1* | -0.32 | 1.89E-11 | 1.99E-10 |
|  | *FSTL1* | 0.76 | 4.08E-79 | 1.72E-76 |  | *AFTPH* | -0.32 | 2.22E-11 | 2.32E-10 |
|  | *ASPN* | 0.76 | 1.31E-78 | 5.40E-76 |  | *ZWINT* | -0.32 | 2.48E-11 | 2.58E-10 |
|  | *TIMP2* | 0.76 | 7.85E-78 | 3.18E-75 |  | *IDH1* | -0.32 | 2.49E-11 | 2.59E-10 |
|  | *LRRC32* | 0.75 | 1.21E-77 | 4.78E-75 |  | *CCNC* | -0.32 | 2.72E-11 | 2.82E-10 |
|  | *PCOLCE* | 0.75 | 1.24E-77 | 4.82E-75 |  | *SNHG10* | -0.32 | 2.98E-11 | 3.07E-10 |
|  | *COL8A2* | 0.75 | 5.54E-77 | 2.12E-74 |  | *RBM25* | -0.32 | 3.39E-11 | 3.47E-10 |
|  | *PLXDC2* | 0.75 | 8.96E-77 | 3.36E-74 |  | *CDS1* | -0.32 | 3.51E-11 | 3.59E-10 |
|  | *RAB31* | 0.75 | 1.05E-76 | 3.87E-74 |  | *PNKD* | -0.32 | 3.88E-11 | 3.95E-10 |
|  | *CCDC80* | 0.75 | 4.20E-76 | 1.52E-73 |  | *AP4B1* | -0.32 | 4.16E-11 | 4.23E-10 |
|  | *INHBA* | 0.75 | 5.98E-76 | 2.12E-73 |  | *CTAGE5* | -0.32 | 4.61E-11 | 4.66E-10 |
|  | *TIMP3* | 0.75 | 6.31E-76 | 2.20E-73 |  | *ATPIF1* | -0.32 | 4.84E-11 | 4.88E-10 |
|  | *FN1* | 0.75 | 7.45E-76 | 2.55E-73 |  | *ERI2* | -0.31 | 5.23E-11 | 5.26E-10 |
|  | *SERPINF1* | 0.75 | 1.72E-75 | 5.81E-73 |  | *C1orf131* | -0.31 | 5.26E-11 | 5.28E-10 |
|  | *SRPX2* | 0.75 | 1.13E-74 | 3.74E-72 |  | *PAPOLA* | -0.31 | 5.30E-11 | 5.32E-10 |
|  | *FAM180A* | 0.74 | 1.76E-74 | 5.73E-72 |  | *MAPK13* | -0.31 | 5.31E-11 | 5.33E-10 |
|  | *FIBIN* | 0.74 | 3.45E-74 | 1.11E-71 |  | *PRSS16* | -0.31 | 5.43E-11 | 5.44E-10 |
|  | *CPZ* | 0.74 | 4.07E-74 | 1.29E-71 |  | *DOM3Z* | -0.31 | 6.19E-11 | 6.17E-10 |
|  | *CLEC11A* | 0.74 | 6.32E-74 | 1.97E-71 |  | *STARD10* | -0.31 | 6.74E-11 | 6.68E-10 |
|  | *MEIS3* | 0.74 | 7.42E-74 | 2.28E-71 |  | *LGALS9B* | -0.31 | 7.26E-11 | 7.17E-10 |
|  | *WISP1* | 0.74 | 9.07E-74 | 2.74E-71 |  | *EPS8L3* | -0.31 | 7.81E-11 | 7.69E-10 |
|  | *TMEM90B* | 0.74 | 3.40E-73 | 1.01E-70 |  | *CSTF3* | -0.31 | 7.98E-11 | 7.84E-10 |
|  | *PCDHGA12* | 0.74 | 4.67E-73 | 1.37E-70 |  | *NDOR1* | -0.31 | 9.04E-11 | 8.84E-10 |
|  | *MXRA8* | 0.74 | 1.25E-72 | 3.62E-70 |  | *ETNK1* | -0.31 | 9.12E-11 | 8.90E-10 |
|  | *TGFB3* | 0.74 | 4.78E-72 | 1.36E-69 |  | *ARHGEF39* | -0.31 | 9.34E-11 | 9.11E-10 |
|  | *ODZ3* | 0.74 | 5.68E-72 | 1.59E-69 |  | *TRIM10* | -0.31 | 9.86E-11 | 9.60E-10 |
|  | *ST6GALNAC5* | 0.74 | 6.19E-72 | 1.72E-69 |  | *ATP5J2* | -0.31 | 1.11E-10 | 1.08E-09 |
|  | *MEDAG* | 0.73 | 1.12E-71 | 3.05E-69 |  | *NOP58* | -0.31 | 1.14E-10 | 1.11E-09 |
|  | *ADAMTS12* | 0.73 | 2.53E-71 | 6.82E-69 |  | *ARPC5L* | -0.31 | 1.19E-10 | 1.15E-09 |
|  | *HTRA3* | 0.73 | 1.30E-70 | 3.45E-68 |  | *CYCS* | -0.31 | 1.27E-10 | 1.22E-09 |
|  | *PODNL1* | 0.73 | 1.37E-70 | 3.60E-68 |  | *RAB11A* | -0.31 | 1.36E-10 | 1.30E-09 |
|  | *EFEMP2* | 0.73 | 2.21E-70 | 5.72E-68 |  | *COX7B* | -0.31 | 1.36E-10 | 1.30E-09 |
|  | *BICC1* | 0.73 | 3.32E-70 | 8.50E-68 |  | *KIAA0101* | -0.31 | 1.38E-10 | 1.32E-09 |
|  | *VSTM4* | 0.73 | 2.08E-69 | 5.27E-67 |  | *ALG6* | -0.31 | 1.42E-10 | 1.36E-09 |
|  | *ANGPTL2* | 0.72 | 1.12E-68 | 2.80E-66 |  | *ESRP1* | -0.31 | 1.44E-10 | 1.37E-09 |
|  | *OLFML1* | 0.72 | 1.28E-68 | 3.15E-66 |  | *OXSM* | -0.31 | 1.44E-10 | 1.37E-09 |
|  | *COL6A2* | 0.72 | 1.58E-68 | 3.85E-66 |  | *FASTKD1* | -0.31 | 1.48E-10 | 1.41E-09 |
|  | *POSTN* | 0.72 | 2.07E-68 | 4.98E-66 |  | *MSH5* | -0.31 | 1.49E-10 | 1.41E-09 |
|  | *CPXM1* | 0.72 | 9.22E-68 | 2.19E-65 |  | *RAB3D* | -0.31 | 1.49E-10 | 1.42E-09 |
|  | *COL12A1* | 0.72 | 1.23E-67 | 2.89E-65 |  | *SPIRE2* | -0.31 | 1.55E-10 | 1.47E-09 |
|  | *SSC5D* | 0.72 | 1.88E-67 | 4.37E-65 |  | *FAM73B* | -0.31 | 1.55E-10 | 1.47E-09 |
|  | *KCND2* | 0.72 | 9.06E-67 | 2.08E-64 |  | *LRPPRC* | -0.31 | 1.58E-10 | 1.49E-09 |
|  | *CERCAM* | 0.72 | 1.34E-66 | 3.05E-64 |  | *ZNF57* | -0.31 | 1.60E-10 | 1.51E-09 |
|  | *DCN* | 0.72 | 1.43E-66 | 3.22E-64 |  | *FOXA3* | -0.31 | 1.73E-10 | 1.63E-09 |
|  | *ARSI* | 0.72 | 3.40E-66 | 7.55E-64 |  | *NSUN5P1* | -0.31 | 1.79E-10 | 1.68E-09 |
|  | *OMD* | 0.71 | 7.65E-66 | 1.68E-63 |  | *ZNF296* | -0.31 | 1.86E-10 | 1.74E-09 |
|  | *DACT1* | 0.71 | 1.39E-64 | 3.03E-62 |  | *RPL36A* | -0.31 | 1.88E-10 | 1.76E-09 |
|  | *COL11A1* | 0.71 | 1.96E-64 | 4.21E-62 |  | *ZNF692* | -0.31 | 1.89E-10 | 1.77E-09 |
|  | *ARMH4* | 0.71 | 2.24E-64 | 4.76E-62 |  | *CBWD3* | -0.31 | 1.92E-10 | 1.80E-09 |
|  | *NALCN* | 0.71 | 2.33E-64 | 4.91E-62 |  | *LRRC1* | -0.31 | 2.04E-10 | 1.91E-09 |
|  | *ISM1* | 0.71 | 5.37E-64 | 1.12E-61 |  | *HMGN1* | -0.31 | 2.12E-10 | 1.97E-09 |
|  | *BNC2* | 0.71 | 5.49E-64 | 1.13E-61 |  | *DDX39* | -0.31 | 2.19E-10 | 2.04E-09 |
|  | *GALNTL2* | 0.71 | 5.70E-64 | 1.16E-61 |  | *TOP1P1* | -0.30 | 2.25E-10 | 2.09E-09 |
|  | *CMTM3* | 0.70 | 4.26E-63 | 8.61E-61 |  | *RTCD1* | -0.30 | 2.35E-10 | 2.18E-09 |
|  | *ADAM12* | 0.70 | 5.49E-63 | 1.10E-60 |  | *ITPKA* | -0.30 | 2.40E-10 | 2.23E-09 |
| *THBS3* | *MAP1A* | 0.76 | 6.28E-80 | 6.35E-76 |  | *RRM2* | -0.55 | 1.63E-34 | 4.59E-33 |
|  | *NFATC4* | 0.74 | 3.92E-74 | 2.28E-70 |  | *CCNA2* | -0.54 | 2.51E-32 | 6.01E-31 |
|  | *SSC5D* | 0.74 | 4.52E-74 | 2.28E-70 |  | *ZWINT* | -0.53 | 9.75E-32 | 2.27E-30 |
|  | *STON1* | 0.72 | 3.44E-68 | 1.39E-64 |  | *CCNB1* | -0.52 | 1.04E-30 | 2.28E-29 |
|  | *MAP3K12* | 0.72 | 4.85E-68 | 1.64E-64 |  | *MAD2L1* | -0.52 | 1.19E-30 | 2.60E-29 |
|  | *ADAM33* | 0.72 | 1.07E-67 | 3.09E-64 |  | *NCAPG* | -0.52 | 1.61E-30 | 3.46E-29 |
|  | *PRELP* | 0.72 | 1.55E-67 | 3.91E-64 |  | *CDC25A* | -0.52 | 1.94E-30 | 4.14E-29 |
|  | *DPYSL3* | 0.72 | 1.29E-66 | 2.90E-63 |  | *PPIF* | -0.52 | 3.33E-30 | 7.02E-29 |
|  | *BNC2* | 0.71 | 9.02E-66 | 1.82E-62 |  | *CKS2* | -0.52 | 3.87E-30 | 8.14E-29 |
|  | *GLI3* | 0.71 | 2.06E-65 | 3.79E-62 |  | *GNPNAT1* | -0.52 | 6.34E-30 | 1.30E-28 |
|  | *FBXL7* | 0.71 | 2.31E-65 | 3.90E-62 |  | *TUBA1C* | -0.51 | 2.35E-29 | 4.64E-28 |
|  | *TRPC1* | 0.71 | 3.44E-65 | 5.35E-62 |  | *ATP5B* | -0.51 | 4.53E-29 | 8.78E-28 |
|  | *NAP1L3* | 0.71 | 5.76E-65 | 8.33E-62 |  | *ATP5G3* | -0.51 | 8.81E-29 | 1.67E-27 |
|  | *VSTM4* | 0.71 | 2.51E-64 | 3.39E-61 |  | *CDCA8* | -0.51 | 8.82E-29 | 1.67E-27 |
|  | *CLIP3* | 0.71 | 4.99E-64 | 6.31E-61 |  | *PTGES3* | -0.51 | 1.72E-28 | 3.20E-27 |
|  | *CCDC80* | 0.71 | 1.15E-63 | 1.37E-60 |  | *SHMT2* | -0.50 | 3.56E-28 | 6.47E-27 |
|  | *MSRB3* | 0.70 | 2.94E-63 | 3.30E-60 |  | *KIAA0101* | -0.50 | 5.29E-28 | 9.50E-27 |
|  | *C14orf132* | 0.70 | 7.17E-63 | 7.63E-60 |  | *GRPEL1* | -0.50 | 6.38E-28 | 1.14E-26 |
|  | *ISM1* | 0.70 | 3.02E-62 | 3.05E-59 |  | *MARS2* | -0.50 | 2.04E-27 | 3.52E-26 |
|  | *PKD2* | 0.70 | 6.80E-62 | 6.52E-59 |  | *PBK* | -0.50 | 2.86E-27 | 4.89E-26 |
|  | *PDLIM3* | 0.70 | 7.10E-62 | 6.52E-59 |  | *SLC25A5* | -0.50 | 3.76E-27 | 6.37E-26 |
|  | *HMCN1* | 0.70 | 1.12E-61 | 9.63E-59 |  | *H2AFZ* | -0.50 | 3.99E-27 | 6.74E-26 |
|  | *PHLDB1* | 0.70 | 1.14E-61 | 9.63E-59 |  | *PPIL5* | -0.49 | 7.20E-27 | 1.20E-25 |
|  | *TNS1* | 0.70 | 1.82E-61 | 1.47E-58 |  | *CCNB2* | -0.49 | 1.14E-26 | 1.88E-25 |
|  | *NACAD* | 0.70 | 2.37E-61 | 1.84E-58 |  | *RAN* | -0.49 | 1.26E-26 | 2.08E-25 |
|  | *MN1* | 0.70 | 2.47E-61 | 1.85E-58 |  | *SFRS9* | -0.49 | 1.31E-26 | 2.16E-25 |
|  | *BOC* | 0.69 | 9.60E-61 | 6.93E-58 |  | *CDK1* | -0.49 | 2.47E-26 | 3.99E-25 |
|  | *TSHZ3* | 0.69 | 1.10E-60 | 7.70E-58 |  | *PTTG1* | -0.49 | 3.39E-26 | 5.44E-25 |
|  | *ZFPM2* | 0.69 | 1.40E-60 | 9.43E-58 |  | *PSMA5* | -0.49 | 3.89E-26 | 6.22E-25 |
|  | *EFEMP2* | 0.69 | 1.79E-60 | 1.16E-57 |  | *KPNA2* | -0.49 | 5.66E-26 | 8.93E-25 |
|  | *GEFT* | 0.69 | 2.85E-60 | 1.80E-57 |  | *MAIP1* | -0.48 | 9.10E-26 | 1.42E-24 |
|  | *PRKD1* | 0.69 | 3.20E-60 | 1.95E-57 |  | *PLK4* | -0.48 | 1.30E-25 | 2.02E-24 |
|  | *FBLN2* | 0.69 | 3.27E-60 | 1.95E-57 |  | *CENPN* | -0.48 | 1.31E-25 | 2.03E-24 |
|  | *ADAMTS10* | 0.69 | 4.22E-60 | 2.44E-57 |  | *COX5A* | -0.48 | 1.33E-25 | 2.06E-24 |
|  | *KANK2* | 0.69 | 7.99E-60 | 4.49E-57 |  | *VRK1* | -0.48 | 1.37E-25 | 2.11E-24 |
|  | *DCLK2* | 0.69 | 9.11E-60 | 4.98E-57 |  | *CYCS* | -0.48 | 1.73E-25 | 2.64E-24 |
|  | *TRO* | 0.69 | 1.53E-59 | 8.17E-57 |  | *SRPK1* | -0.48 | 2.89E-25 | 4.39E-24 |
|  | *EVC* | 0.69 | 1.86E-59 | 9.66E-57 |  | *UBE2T* | -0.48 | 3.65E-25 | 5.51E-24 |
|  | *ZCCHC24* | 0.69 | 2.32E-59 | 1.17E-56 |  | *MTHFD2* | -0.48 | 3.95E-25 | 5.93E-24 |
|  | *COL8A1* | 0.69 | 2.72E-59 | 1.34E-56 |  | *HSPD1* | -0.48 | 4.24E-25 | 6.36E-24 |
|  | *LOC399959* | 0.69 | 3.88E-59 | 1.84E-56 |  | *PARPBP* | -0.48 | 4.78E-25 | 7.13E-24 |
|  | *CALD1* | 0.69 | 3.91E-59 | 1.84E-56 |  | *ORC6L* | -0.48 | 5.94E-25 | 8.82E-24 |
|  | *FSTL1* | 0.69 | 4.09E-59 | 1.88E-56 |  | *MRPL44* | -0.47 | 1.37E-24 | 1.99E-23 |
|  | *SCARF2* | 0.69 | 8.91E-59 | 4.01E-56 |  | *POC1A* | -0.47 | 1.59E-24 | 2.30E-23 |
|  | *ZNF423* | 0.68 | 1.28E-58 | 5.63E-56 |  | *ORC1L* | -0.47 | 1.87E-24 | 2.69E-23 |
|  | *TGFB1I1* | 0.68 | 1.34E-58 | 5.75E-56 |  | *ASF1B* | -0.47 | 2.07E-24 | 2.98E-23 |
|  | *PTRF* | 0.68 | 2.05E-58 | 8.65E-56 |  | *HNRNPC* | -0.47 | 2.25E-24 | 3.23E-23 |
|  | *GLT8D2* | 0.68 | 2.55E-58 | 1.05E-55 |  | *SLBP* | -0.47 | 2.99E-24 | 4.26E-23 |
|  | *DACT1* | 0.68 | 3.49E-58 | 1.41E-55 |  | *FAM54A* | -0.47 | 3.60E-24 | 5.10E-23 |
|  | *MRGPRF* | 0.68 | 4.74E-58 | 1.88E-55 |  | *FEN1* | -0.47 | 3.87E-24 | 5.47E-23 |
|  | *PRICKLE2* | 0.68 | 9.00E-58 | 3.50E-55 |  | *NUP37* | -0.47 | 4.84E-24 | 6.80E-23 |
|  | *PODN* | 0.68 | 9.66E-58 | 3.68E-55 |  | *C1QBP* | -0.47 | 5.32E-24 | 7.43E-23 |
|  | *CNTNAP1* | 0.68 | 1.02E-57 | 3.81E-55 |  | *MRPS35* | -0.47 | 5.50E-24 | 7.67E-23 |
|  | *EFS* | 0.68 | 1.40E-57 | 5.13E-55 |  | *AURKA* | -0.47 | 5.73E-24 | 7.97E-23 |
|  | *DZIP1* | 0.68 | 1.72E-57 | 6.22E-55 |  | *PA2G4* | -0.47 | 7.17E-24 | 9.93E-23 |
|  | *COL6A2* | 0.68 | 1.76E-57 | 6.26E-55 |  | *AIFM1* | -0.47 | 9.61E-24 | 1.32E-22 |
|  | *MAGI2* | 0.68 | 2.52E-57 | 8.78E-55 |  | *CDC20* | -0.47 | 1.05E-23 | 1.44E-22 |
|  | *NDN* | 0.68 | 3.31E-57 | 1.13E-54 |  | *KIF18A* | -0.46 | 1.19E-23 | 1.62E-22 |
|  | *CSDC2* | 0.68 | 3.44E-57 | 1.16E-54 |  | *PLK1* | -0.46 | 1.22E-23 | 1.66E-22 |
|  | *FAM180A* | 0.68 | 3.60E-57 | 1.19E-54 |  | *RACGAP1* | -0.46 | 1.33E-23 | 1.80E-22 |
|  | *SYDE1* | 0.68 | 3.94E-57 | 1.29E-54 |  | *TCP1* | -0.46 | 1.65E-23 | 2.20E-22 |
|  | *RUNX1T1* | 0.68 | 5.01E-57 | 1.61E-54 |  | *DKC1* | -0.46 | 2.25E-23 | 2.97E-22 |
|  | *OMD* | 0.68 | 5.45E-57 | 1.72E-54 |  | *ESCO2* | -0.46 | 2.32E-23 | 3.06E-22 |
|  | *ZNF521* | 0.68 | 5.85E-57 | 1.82E-54 |  | *CDC45* | -0.46 | 2.34E-23 | 3.09E-22 |
|  | *PEG3* | 0.68 | 6.55E-57 | 2.01E-54 |  | *CCT2* | -0.46 | 2.46E-23 | 3.23E-22 |
|  | *ZFHX4* | 0.68 | 8.93E-57 | 2.70E-54 |  | *GINS2* | -0.46 | 3.14E-23 | 4.11E-22 |
|  | *LHFP* | 0.68 | 1.14E-56 | 3.38E-54 |  | *OIP5* | -0.46 | 3.23E-23 | 4.22E-22 |
|  | *TIMP2* | 0.68 | 1.18E-56 | 3.47E-54 |  | *MCCC2* | -0.46 | 3.36E-23 | 4.39E-22 |
|  | *COL8A2* | 0.68 | 1.55E-56 | 4.47E-54 |  | *MELK* | -0.46 | 3.83E-23 | 4.97E-22 |
|  | *AEBP1* | 0.67 | 2.08E-56 | 5.93E-54 |  | *NUSAP1* | -0.46 | 4.24E-23 | 5.48E-22 |
|  | *DCN* | 0.67 | 2.26E-56 | 6.35E-54 |  | *PAICS* | -0.46 | 4.37E-23 | 5.63E-22 |
|  | *ISLR* | 0.67 | 3.99E-56 | 1.11E-53 |  | *CHAC2* | -0.46 | 4.57E-23 | 5.87E-22 |
|  | *INMT* | 0.67 | 4.66E-56 | 1.27E-53 |  | *NDUFA9* | -0.46 | 4.59E-23 | 5.90E-22 |
|  | *PLSCR4* | 0.67 | 4.72E-56 | 1.27E-53 |  | *SGOL1* | -0.46 | 5.25E-23 | 6.71E-22 |
|  | *FGFR1* | 0.67 | 4.79E-56 | 1.27E-53 |  | *RFC5* | -0.46 | 5.90E-23 | 7.51E-22 |
|  | *SFRP4* | 0.67 | 6.64E-56 | 1.74E-53 |  | *LRRC59* | -0.46 | 6.45E-23 | 8.17E-22 |
|  | *COL14A1* | 0.67 | 7.27E-56 | 1.88E-53 |  | *GMNN* | -0.46 | 7.01E-23 | 8.85E-22 |
|  | *JAM3* | 0.67 | 1.08E-55 | 2.76E-53 |  | *BZW2* | -0.46 | 9.56E-23 | 1.20E-21 |
|  | *POU6F1* | 0.67 | 1.70E-55 | 4.31E-53 |  | *DEPDC1B* | -0.46 | 1.00E-22 | 1.25E-21 |
|  | *MARVELD1* | 0.67 | 1.77E-55 | 4.38E-53 |  | *SFRS1* | -0.45 | 1.75E-22 | 2.13E-21 |
|  | *ZNF135* | 0.67 | 1.78E-55 | 4.38E-53 |  | *POLE2* | -0.45 | 2.26E-22 | 2.73E-21 |
|  | *FBLN1* | 0.67 | 2.39E-55 | 5.80E-53 |  | *MND1* | -0.45 | 2.33E-22 | 2.81E-21 |
|  | *DACT3* | 0.67 | 2.41E-55 | 5.80E-53 |  | *SFRS2* | -0.45 | 2.35E-22 | 2.83E-21 |
|  | *OLFML1* | 0.67 | 3.08E-55 | 7.34E-53 |  | *BIRC5* | -0.45 | 2.41E-22 | 2.91E-21 |
|  | *GUCY1A3* | 0.67 | 3.25E-55 | 7.65E-53 |  | *ERP44* | -0.45 | 2.63E-22 | 3.15E-21 |
|  | *PABPC5* | 0.67 | 3.86E-55 | 8.97E-53 |  | *CCNC* | -0.45 | 3.13E-22 | 3.75E-21 |
|  | *SFRP2* | 0.67 | 4.77E-55 | 1.10E-52 |  | *RAD51AP1* | -0.45 | 3.82E-22 | 4.56E-21 |
|  | *MRAS* | 0.67 | 4.88E-55 | 1.11E-52 |  | *SPC25* | -0.45 | 4.21E-22 | 5.00E-21 |
|  | *CDO1* | 0.67 | 5.13E-55 | 1.15E-52 |  | *SFT2D1* | -0.45 | 4.72E-22 | 5.60E-21 |
|  | *FBN1* | 0.67 | 5.52E-55 | 1.23E-52 |  | *AMD1* | -0.45 | 5.29E-22 | 6.23E-21 |
|  | *MPDZ* | 0.67 | 6.10E-55 | 1.34E-52 |  | *MRPS30* | -0.45 | 6.02E-22 | 7.06E-21 |
|  | *MRVI1* | 0.67 | 7.00E-55 | 1.52E-52 |  | *DLGAP5* | -0.45 | 6.48E-22 | 7.59E-21 |
|  | *COL16A1* | 0.67 | 7.49E-55 | 1.61E-52 |  | *MRPL3* | -0.45 | 6.93E-22 | 8.10E-21 |
|  | *GPR124* | 0.67 | 8.72E-55 | 1.86E-52 |  | *MYCBP* | -0.45 | 7.63E-22 | 8.89E-21 |
|  | *ECM2* | 0.67 | 9.33E-55 | 1.97E-52 |  | *EZH2* | -0.45 | 7.81E-22 | 9.09E-21 |
|  | *SLIT2* | 0.67 | 1.07E-54 | 2.23E-52 |  | *NME1* | -0.45 | 9.00E-22 | 1.04E-20 |
|  | *LIMS2* | 0.67 | 1.40E-54 | 2.89E-52 |  | *CDCA2* | -0.45 | 1.09E-21 | 1.25E-20 |
|  | *SLC45A1* | 0.67 | 2.15E-54 | 4.39E-52 |  | *CENPK* | -0.45 | 1.09E-21 | 1.25E-20 |
|  | *AOC3* | 0.66 | 2.65E-54 | 5.36E-52 |  | *CDCA5* | -0.45 | 1.16E-21 | 1.34E-20 |
|  | *ASPN* | 0.65 | 5.49E-51 | 7.12E-49 |  | *CENPA* | -0.45 | 1.22E-21 | 1.40E-20 |
| *THBS4* | *BOC* | 0.89 | 2.06E-139 | 2.08E-135 |  | *NCAPG* | -0.54 | 1.42E-32 | 2.29E-31 |
|  | *CCDC80* | 0.86 | 4.38E-122 | 2.95E-118 |  | *MELK* | -0.51 | 3.59E-29 | 4.96E-28 |
|  | *AOC3* | 0.86 | 2.14E-120 | 1.08E-116 |  | *DKC1* | -0.51 | 3.65E-29 | 5.03E-28 |
|  | *PRELP* | 0.86 | 6.48E-120 | 2.62E-116 |  | *CDCA8* | -0.51 | 4.54E-29 | 6.23E-28 |
|  | *ANGPTL1* | 0.85 | 2.84E-119 | 9.59E-116 |  | *MAD2L1* | -0.51 | 1.55E-28 | 2.07E-27 |
|  | *MGP* | 0.85 | 1.09E-117 | 3.14E-114 |  | *RAD51AP1* | -0.50 | 6.57E-28 | 8.53E-27 |
|  | *CILP* | 0.85 | 8.07E-117 | 2.04E-113 |  | *ZWINT* | -0.50 | 7.72E-28 | 9.98E-27 |
|  | *CYP1B1* | 0.85 | 1.66E-116 | 3.73E-113 |  | *KPNA2* | -0.50 | 1.02E-27 | 1.31E-26 |
|  | *SLIT2* | 0.85 | 3.31E-116 | 6.69E-113 |  | *HOOK1* | -0.50 | 1.66E-27 | 2.11E-26 |
|  | *OGN* | 0.85 | 7.00E-116 | 1.29E-112 |  | *PLK4* | -0.50 | 1.70E-27 | 2.16E-26 |
|  | *ZCCHC24* | 0.84 | 7.31E-113 | 1.23E-109 |  | *CDK1* | -0.50 | 1.94E-27 | 2.46E-26 |
|  | *GUCY1A3* | 0.84 | 8.36E-112 | 1.30E-108 |  | *SNRPA1* | -0.50 | 4.63E-27 | 5.78E-26 |
|  | *PDLIM3* | 0.84 | 6.22E-111 | 8.98E-108 |  | *SPAG5* | -0.49 | 4.99E-27 | 6.21E-26 |
|  | *LOC399959* | 0.84 | 1.09E-110 | 1.47E-107 |  | *SLC25A10* | -0.49 | 8.95E-27 | 1.10E-25 |
|  | *CDO1* | 0.84 | 5.88E-110 | 7.43E-107 |  | *KIAA0101* | -0.49 | 1.43E-26 | 1.74E-25 |
|  | *RGMA* | 0.83 | 4.46E-109 | 5.31E-106 |  | *MAP7* | -0.49 | 1.69E-26 | 2.05E-25 |
|  | *CHRDL1* | 0.83 | 1.04E-108 | 1.17E-105 |  | *SRPK1* | -0.49 | 2.46E-26 | 2.97E-25 |
|  | *PLN* | 0.83 | 5.13E-108 | 5.46E-105 |  | *AURKA* | -0.49 | 2.97E-26 | 3.57E-25 |
|  | *LMOD1* | 0.83 | 7.24E-108 | 7.32E-105 |  | *TOP2A* | -0.49 | 4.57E-26 | 5.46E-25 |
|  | *PTGIS* | 0.83 | 1.48E-106 | 1.42E-103 |  | *TRAF4* | -0.49 | 5.54E-26 | 6.59E-25 |
|  | *CPXM2* | 0.83 | 7.85E-106 | 7.22E-103 |  | *HSP90AB1* | -0.49 | 5.73E-26 | 6.80E-25 |
|  | *DPYSL3* | 0.83 | 1.78E-105 | 1.57E-102 |  | *MYO19* | -0.49 | 5.94E-26 | 7.03E-25 |
|  | *HSPB7* | 0.83 | 4.65E-105 | 3.92E-102 |  | *UBE2T* | -0.49 | 6.75E-26 | 7.97E-25 |
|  | *TNS1* | 0.83 | 9.10E-105 | 7.36E-102 |  | *MYCBP* | -0.48 | 7.22E-26 | 8.50E-25 |
|  | *ABCC9* | 0.82 | 4.46E-104 | 3.47E-101 |  | *SKA3* | -0.48 | 7.62E-26 | 8.95E-25 |
|  | *RUNX1T1* | 0.82 | 5.53E-104 | 4.14E-101 |  | *ANLN* | -0.48 | 1.32E-25 | 1.54E-24 |
|  | *RNF150* | 0.82 | 8.26E-104 | 5.97E-101 |  | *RRM2* | -0.48 | 1.38E-25 | 1.61E-24 |
|  | *CLIP3* | 0.82 | 1.04E-103 | 7.28E-101 |  | *NCAPG2* | -0.48 | 2.24E-25 | 2.57E-24 |
|  | *SFRP1* | 0.82 | 1.60E-103 | 1.08E-100 |  | *HSPD1* | -0.48 | 3.75E-25 | 4.23E-24 |
|  | *PGM5* | 0.82 | 4.81E-103 | 3.14E-100 |  | *ZC3H12A* | -0.48 | 3.94E-25 | 4.44E-24 |
|  | *MEOX2* | 0.82 | 2.05E-102 | 1.30E-99 |  | *HNRNPA2B1* | -0.48 | 6.12E-25 | 6.86E-24 |
|  | *NAP1L3* | 0.82 | 3.28E-102 | 2.01E-99 |  | *FANCI* | -0.48 | 8.15E-25 | 9.10E-24 |
|  | *MYLK* | 0.82 | 4.71E-102 | 2.80E-99 |  | *ARHGEF39* | -0.48 | 8.55E-25 | 9.53E-24 |
|  | *HSPB6* | 0.82 | 4.92E-102 | 2.84E-99 |  | *CDC6* | -0.48 | 8.81E-25 | 9.80E-24 |
|  | *PODN* | 0.82 | 1.03E-101 | 5.78E-99 |  | *CDC25A* | -0.48 | 9.17E-25 | 1.02E-23 |
|  | *MSRB3* | 0.82 | 1.90E-101 | 1.04E-98 |  | *CKS1B* | -0.47 | 1.09E-24 | 1.20E-23 |
|  | *STON1* | 0.82 | 2.65E-101 | 1.41E-98 |  | *BUB1B* | -0.47 | 1.20E-24 | 1.32E-23 |
|  | *TAGLN* | 0.82 | 1.30E-100 | 6.75E-98 |  | *POLQ* | -0.47 | 1.50E-24 | 1.64E-23 |
|  | *DACT3* | 0.82 | 3.48E-100 | 1.76E-97 |  | *GNL3* | -0.47 | 1.69E-24 | 1.85E-23 |
|  | *BNC2* | 0.81 | 7.02E-100 | 3.46E-97 |  | *BZW2* | -0.47 | 1.71E-24 | 1.87E-23 |
|  | *SCRG1* | 0.81 | 9.04E-100 | 4.35E-97 |  | *KIF18A* | -0.47 | 1.95E-24 | 2.13E-23 |
|  | *INMT* | 0.81 | 1.37E-99 | 6.46E-97 |  | *CNOT11* | -0.47 | 2.45E-24 | 2.66E-23 |
|  | *SYNPO2* | 0.81 | 1.69E-99 | 7.77E-97 |  | *CCNB2* | -0.47 | 3.36E-24 | 3.63E-23 |
|  | *MYL9* | 0.81 | 4.20E-98 | 1.89E-95 |  | *CDC45* | -0.47 | 3.66E-24 | 3.94E-23 |
|  | *CNN1* | 0.81 | 1.36E-97 | 5.96E-95 |  | *HMMR* | -0.47 | 3.85E-24 | 4.15E-23 |
|  | *GREM1* | 0.81 | 3.66E-97 | 1.58E-94 |  | *ESRP1* | -0.47 | 4.66E-24 | 5.01E-23 |
|  | *KCNMA1* | 0.81 | 4.44E-97 | 1.87E-94 |  | *RAD54L* | -0.47 | 6.20E-24 | 6.62E-23 |
|  | *OMD* | 0.81 | 8.93E-97 | 3.69E-94 |  | *DLGAP5* | -0.47 | 9.31E-24 | 9.86E-23 |
|  | *ATP1A2* | 0.81 | 5.40E-96 | 2.19E-93 |  | *PSMD14* | -0.47 | 9.75E-24 | 1.03E-22 |
|  | *COL8A1* | 0.80 | 2.79E-95 | 1.11E-92 |  | *CCNA2* | -0.47 | 9.89E-24 | 1.05E-22 |
|  | *HAND2* | 0.80 | 3.68E-95 | 1.43E-92 |  | *SFRS1* | -0.47 | 1.05E-23 | 1.11E-22 |
|  | *VSTM4* | 0.80 | 7.36E-95 | 2.81E-92 |  | *RECQL4* | -0.47 | 1.12E-23 | 1.19E-22 |
|  | *CASQ2* | 0.80 | 9.41E-95 | 3.52E-92 |  | *PARPBP* | -0.46 | 1.43E-23 | 1.50E-22 |
|  | *RSPO3* | 0.80 | 1.16E-94 | 4.25E-92 |  | *MARS2* | -0.46 | 1.73E-23 | 1.81E-22 |
|  | *NRK* | 0.80 | 2.22E-94 | 8.02E-92 |  | *RFC3* | -0.46 | 1.75E-23 | 1.82E-22 |
|  | *DDR2* | 0.80 | 1.93E-93 | 6.85E-91 |  | *PRC1* | -0.46 | 1.88E-23 | 1.96E-22 |
|  | *PDZRN4* | 0.80 | 2.51E-93 | 8.75E-91 |  | *TPX2* | -0.46 | 2.33E-23 | 2.42E-22 |
|  | *NDN* | 0.80 | 2.62E-93 | 8.97E-91 |  | *HJURP* | -0.46 | 2.36E-23 | 2.45E-22 |
|  | *ZFPM2* | 0.80 | 2.75E-93 | 9.28E-91 |  | *CENPA* | -0.46 | 2.47E-23 | 2.57E-22 |
|  | *PABPC5* | 0.80 | 5.48E-93 | 1.82E-90 |  | *PAICS* | -0.46 | 2.77E-23 | 2.87E-22 |
|  | *FHL1* | 0.80 | 8.90E-93 | 2.90E-90 |  | *EZH2* | -0.46 | 3.11E-23 | 3.22E-22 |
|  | *FXYD6* | 0.80 | 1.71E-92 | 5.49E-90 |  | *ASF1B* | -0.46 | 4.15E-23 | 4.26E-22 |
|  | *JAM2* | 0.80 | 2.36E-92 | 7.45E-90 |  | *FEN1* | -0.46 | 4.35E-23 | 4.46E-22 |
|  | *SSC5D* | 0.80 | 2.62E-92 | 8.17E-90 |  | *KIF15* | -0.46 | 4.51E-23 | 4.62E-22 |
|  | *ACTA2* | 0.80 | 2.74E-92 | 8.38E-90 |  | *SLMO2* | -0.46 | 4.89E-23 | 5.01E-22 |
|  | *MRGPRF* | 0.80 | 4.88E-92 | 1.47E-89 |  | *DBF4* | -0.46 | 4.96E-23 | 5.07E-22 |
|  | *NBLA00301* | 0.80 | 8.19E-92 | 2.43E-89 |  | *RACGAP1* | -0.46 | 5.20E-23 | 5.30E-22 |
|  | *GEFT* | 0.80 | 9.76E-92 | 2.86E-89 |  | *KIF23* | -0.46 | 5.69E-23 | 5.79E-22 |
|  | *ANK2* | 0.79 | 1.16E-91 | 3.36E-89 |  | *FIGNL1* | -0.46 | 5.92E-23 | 6.02E-22 |
|  | *EFEMP1* | 0.79 | 2.16E-91 | 6.17E-89 |  | *KDF1* | -0.46 | 6.46E-23 | 6.56E-22 |
|  | *MAP1A* | 0.79 | 5.64E-91 | 1.58E-88 |  | *FAM54A* | -0.46 | 1.13E-22 | 1.13E-21 |
|  | *SPARCL1* | 0.79 | 6.45E-91 | 1.79E-88 |  | *NCAPH* | -0.46 | 1.22E-22 | 1.22E-21 |
|  | *FLRT2* | 0.79 | 1.69E-90 | 4.62E-88 |  | *C6orf136* | -0.46 | 1.25E-22 | 1.25E-21 |
|  | *C7* | 0.79 | 2.24E-90 | 6.03E-88 |  | *EME1* | -0.45 | 1.44E-22 | 1.43E-21 |
|  | *SCN7A* | 0.79 | 2.87E-90 | 7.64E-88 |  | *E2F8* | -0.45 | 1.64E-22 | 1.63E-21 |
|  | *PDZD4* | 0.79 | 3.29E-90 | 8.65E-88 |  | *ATAD2* | -0.45 | 1.87E-22 | 1.85E-21 |
|  | *GUCY1B3* | 0.79 | 3.53E-90 | 9.15E-88 |  | *MTHFD2* | -0.45 | 1.90E-22 | 1.87E-21 |
|  | *DCLK1* | 0.79 | 6.26E-90 | 1.60E-87 |  | *PLK1* | -0.45 | 1.92E-22 | 1.90E-21 |
|  | *LOC339524* | 0.79 | 8.13E-90 | 2.06E-87 |  | *AP1G2* | -0.45 | 1.93E-22 | 1.90E-21 |
|  | *PDE1A* | 0.79 | 9.41E-90 | 2.35E-87 |  | *DHX15* | -0.45 | 1.98E-22 | 1.95E-21 |
|  | *GNAO1* | 0.79 | 1.65E-89 | 4.07E-87 |  | *CKS2* | -0.45 | 2.12E-22 | 2.08E-21 |
|  | *MYH11* | 0.79 | 1.78E-89 | 4.33E-87 |  | *SASS6* | -0.45 | 2.30E-22 | 2.26E-21 |
|  | *LAYN* | 0.79 | 2.36E-89 | 5.67E-87 |  | *ENOPH1* | -0.45 | 3.21E-22 | 3.14E-21 |
|  | *ADAMTSL3* | 0.79 | 2.98E-89 | 7.10E-87 |  | *GNPNAT1* | -0.45 | 4.38E-22 | 4.26E-21 |
|  | *MAPK10* | 0.79 | 3.74E-89 | 8.79E-87 |  | *LRRC1* | -0.45 | 4.66E-22 | 4.52E-21 |
|  | *PTGER3* | 0.79 | 1.30E-88 | 3.03E-86 |  | *TRIP13* | -0.45 | 4.99E-22 | 4.83E-21 |
|  | *FGF7* | 0.79 | 2.19E-88 | 5.04E-86 |  | *KIF2C* | -0.45 | 5.54E-22 | 5.34E-21 |
|  | *HMCN1* | 0.79 | 4.23E-88 | 9.62E-86 |  | *PBK* | -0.45 | 5.73E-22 | 5.52E-21 |
|  | *MRAS* | 0.78 | 8.76E-88 | 1.97E-85 |  | *FAM72B* | -0.45 | 6.46E-22 | 6.20E-21 |
|  | *CHRDL2* | 0.78 | 1.39E-87 | 3.09E-85 |  | *AP1AR* | -0.45 | 6.58E-22 | 6.31E-21 |
|  | *PLP1* | 0.78 | 3.94E-87 | 8.65E-85 |  | *KIF4A* | -0.45 | 6.64E-22 | 6.36E-21 |
|  | *MRVI1* | 0.78 | 4.18E-87 | 9.09E-85 |  | *ESPL1* | -0.45 | 6.84E-22 | 6.55E-21 |
|  | *CALD1* | 0.78 | 2.84E-86 | 6.10E-84 |  | *CMTM8* | -0.45 | 6.84E-22 | 6.55E-21 |
|  | *COL14A1* | 0.78 | 3.96E-86 | 8.44E-84 |  | *SHCBP1* | -0.45 | 7.15E-22 | 6.83E-21 |
|  | *PCDH10* | 0.78 | 4.99E-86 | 1.05E-83 |  | *EPT1* | -0.45 | 7.88E-22 | 7.50E-21 |
|  | *JAM3* | 0.78 | 1.10E-85 | 2.30E-83 |  | *TBRG4* | -0.45 | 8.47E-22 | 8.04E-21 |
|  | *IGF1* | 0.78 | 1.68E-85 | 3.47E-83 |  | *CDCA5* | -0.45 | 8.78E-22 | 8.33E-21 |
|  | *MPDZ* | 0.78 | 6.17E-85 | 1.26E-82 |  | *SGOL1* | -0.45 | 9.82E-22 | 9.29E-21 |
|  | *PEG3* | 0.78 | 7.85E-85 | 1.59E-82 |  | *STIL* | -0.45 | 1.05E-21 | 9.88E-21 |
|  | *LGI2* | 0.78 | 1.46E-84 | 2.92E-82 |  | *BIRC5* | -0.45 | 1.07E-21 | 1.01E-20 |
| *COMP* | *ITGBL1* | 0.73 | 1.49E-71 | 1.51E-67 |  | *GNPNAT1* | -0.42 | 2.56E-19 | 6.26E-18 |
|  | *SFRP4* | 0.73 | 7.34E-71 | 4.95E-67 |  | *TC2N* | -0.41 | 3.68E-18 | 7.86E-17 |
|  | *FNDC1* | 0.70 | 2.99E-62 | 1.51E-58 |  | *GSKIP* | -0.41 | 4.55E-18 | 9.62E-17 |
|  | *NALCN* | 0.68 | 1.42E-58 | 5.72E-55 |  | *LRPPRC* | -0.39 | 6.34E-17 | 1.20E-15 |
|  | *COL8A1* | 0.68 | 6.44E-58 | 2.17E-54 |  | *SRPK1* | -0.39 | 6.91E-17 | 1.30E-15 |
|  | *COL8A2* | 0.67 | 2.43E-55 | 7.03E-52 |  | *RBM47* | -0.39 | 2.95E-16 | 5.31E-15 |
|  | *BGN* | 0.67 | 2.09E-54 | 5.28E-51 |  | *MYCBP* | -0.38 | 9.94E-16 | 1.70E-14 |
|  | *FGF14* | 0.66 | 4.76E-54 | 1.07E-50 |  | *AP4B1* | -0.38 | 1.13E-15 | 1.92E-14 |
|  | *AEBP1* | 0.66 | 5.42E-53 | 1.10E-49 |  | *MST1R* | -0.37 | 4.75E-15 | 7.61E-14 |
|  | *MXRA8* | 0.66 | 6.08E-53 | 1.12E-49 |  | *AP1AR* | -0.37 | 5.90E-15 | 9.35E-14 |
|  | *GXYLT2* | 0.66 | 2.51E-52 | 4.22E-49 |  | *PI4K2B* | -0.37 | 8.37E-15 | 1.30E-13 |
|  | *ISLR* | 0.65 | 6.69E-52 | 1.04E-48 |  | *SLC35A3* | -0.37 | 8.46E-15 | 1.31E-13 |
|  | *SFRP2* | 0.65 | 8.71E-52 | 1.26E-48 |  | *MST4* | -0.37 | 9.68E-15 | 1.50E-13 |
|  | *MFAP5* | 0.65 | 2.56E-50 | 3.45E-47 |  | *DDAH1* | -0.37 | 1.05E-14 | 1.62E-13 |
|  | *TIMP3* | 0.64 | 3.67E-50 | 4.64E-47 |  | *GRSF1* | -0.36 | 1.82E-14 | 2.76E-13 |
|  | *SCARF2* | 0.64 | 8.07E-50 | 9.60E-47 |  | *LRRC1* | -0.36 | 2.10E-14 | 3.16E-13 |
|  | *CDH11* | 0.64 | 3.57E-49 | 4.02E-46 |  | *BZW2* | -0.36 | 3.22E-14 | 4.77E-13 |
|  | *PRELP* | 0.64 | 7.30E-49 | 7.77E-46 |  | *ERI2* | -0.36 | 3.65E-14 | 5.34E-13 |
|  | *FBLN2* | 0.64 | 1.14E-48 | 1.15E-45 |  | *GUF1* | -0.36 | 4.73E-14 | 6.87E-13 |
|  | *LTBP2* | 0.64 | 1.20E-48 | 1.15E-45 |  | *GNL3* | -0.36 | 5.04E-14 | 7.30E-13 |
|  | *EFEMP2* | 0.64 | 1.27E-48 | 1.17E-45 |  | *FASTKD1* | -0.36 | 5.19E-14 | 7.49E-13 |
|  | *THBS2* | 0.64 | 3.05E-48 | 2.68E-45 |  | *NCAPG* | -0.36 | 7.81E-14 | 1.11E-12 |
|  | *ITGA11* | 0.63 | 1.10E-47 | 9.29E-45 |  | *CCNK* | -0.36 | 8.69E-14 | 1.22E-12 |
|  | *P4HA3* | 0.63 | 1.23E-47 | 9.92E-45 |  | *PDE12* | -0.35 | 1.16E-13 | 1.62E-12 |
|  | *CCDC8* | 0.63 | 3.04E-47 | 2.37E-44 |  | *MARS2* | -0.35 | 1.38E-13 | 1.91E-12 |
|  | *ISM1* | 0.63 | 3.70E-47 | 2.77E-44 |  | *CD2AP* | -0.35 | 1.38E-13 | 1.91E-12 |
|  | *FAM180A* | 0.63 | 5.21E-47 | 3.76E-44 |  | *PPA2* | -0.35 | 1.56E-13 | 2.14E-12 |
|  | *FBLN1* | 0.63 | 5.53E-47 | 3.85E-44 |  | *CDS1* | -0.35 | 1.66E-13 | 2.27E-12 |
|  | *HMCN1* | 0.63 | 7.37E-47 | 4.97E-44 |  | *RRM2* | -0.35 | 1.85E-13 | 2.52E-12 |
|  | *ANTXR1* | 0.62 | 3.05E-46 | 1.99E-43 |  | *PLEKHH1* | -0.35 | 2.21E-13 | 3.00E-12 |
|  | *CTHRC1* | 0.62 | 4.53E-46 | 2.87E-43 |  | *TWF1* | -0.35 | 2.85E-13 | 3.82E-12 |
|  | *MAGEL2* | 0.62 | 6.53E-46 | 4.00E-43 |  | *UBA6* | -0.35 | 4.24E-13 | 5.59E-12 |
|  | *MATN3* | 0.62 | 2.01E-45 | 1.19E-42 |  | *PCSK7* | -0.35 | 4.27E-13 | 5.62E-12 |
|  | *SUGCT* | 0.62 | 2.80E-45 | 1.62E-42 |  | *TMEM161B* | -0.34 | 7.59E-13 | 9.72E-12 |
|  | *SERPINF1* | 0.62 | 6.41E-45 | 3.60E-42 |  | *ABCD3* | -0.34 | 8.33E-13 | 1.06E-11 |
|  | *FGFR1* | 0.62 | 9.13E-45 | 4.99E-42 |  | *CCBL2* | -0.34 | 8.42E-13 | 1.07E-11 |
|  | *FXYD6* | 0.62 | 1.06E-44 | 5.62E-42 |  | *TPMT* | -0.34 | 1.19E-12 | 1.49E-11 |
|  | *NTM* | 0.62 | 1.16E-44 | 6.00E-42 |  | *EPT1* | -0.34 | 1.41E-12 | 1.75E-11 |
|  | *ELN* | 0.61 | 2.92E-44 | 1.47E-41 |  | *CHAC2* | -0.34 | 2.07E-12 | 2.52E-11 |
|  | *MN1* | 0.61 | 4.27E-44 | 2.08E-41 |  | *HNRNPA2B1* | -0.34 | 2.10E-12 | 2.55E-11 |
|  | *PTGIS* | 0.61 | 4.33E-44 | 2.08E-41 |  | *PBK* | -0.34 | 2.41E-12 | 2.90E-11 |
|  | *PODN* | 0.61 | 8.90E-44 | 4.19E-41 |  | *KIAA1468* | -0.33 | 3.50E-12 | 4.15E-11 |
|  | *NAP1L3* | 0.61 | 1.79E-43 | 8.21E-41 |  | *FAM120A* | -0.33 | 3.95E-12 | 4.67E-11 |
|  | *COPZ2* | 0.61 | 1.83E-43 | 8.21E-41 |  | *CCDC68* | -0.33 | 4.08E-12 | 4.81E-11 |
|  | *MFRP* | 0.61 | 2.90E-43 | 1.27E-40 |  | *MTIF2* | -0.33 | 4.27E-12 | 5.03E-11 |
|  | *SLIT2* | 0.61 | 3.20E-43 | 1.38E-40 |  | *EZH2* | -0.33 | 7.28E-12 | 8.31E-11 |
|  | *EFEMP1* | 0.61 | 8.65E-43 | 3.64E-40 |  | *CHP* | -0.33 | 8.35E-12 | 9.48E-11 |
|  | *OMD* | 0.60 | 8.81E-43 | 3.64E-40 |  | *CXCL3* | -0.33 | 9.93E-12 | 1.12E-10 |
|  | *MFAP2* | 0.60 | 1.08E-42 | 4.35E-40 |  | *MRPS35* | -0.33 | 1.01E-11 | 1.13E-10 |
|  | *SULF1* | 0.60 | 2.34E-42 | 9.28E-40 |  | *MYO5C* | -0.33 | 1.02E-11 | 1.15E-10 |
|  | *GFPT2* | 0.60 | 3.49E-42 | 1.36E-39 |  | *BCL10* | -0.33 | 1.05E-11 | 1.17E-10 |
|  | *SPOCK1* | 0.60 | 4.31E-42 | 1.64E-39 |  | *ESRP2* | -0.33 | 1.11E-11 | 1.24E-10 |
|  | *CLEC11A* | 0.60 | 5.70E-42 | 2.12E-39 |  | *E2F8* | -0.32 | 1.37E-11 | 1.52E-10 |
|  | *DACT1* | 0.60 | 5.77E-42 | 2.12E-39 |  | *GLMN* | -0.32 | 1.65E-11 | 1.81E-10 |
|  | *CMTM3* | 0.60 | 1.01E-41 | 3.66E-39 |  | *TRIM40* | -0.32 | 1.87E-11 | 2.06E-10 |
|  | *SPARC* | 0.60 | 1.46E-41 | 5.17E-39 |  | *DHX15* | -0.32 | 1.89E-11 | 2.07E-10 |
|  | *CPZ* | 0.60 | 1.49E-41 | 5.18E-39 |  | *DBF4* | -0.32 | 1.99E-11 | 2.18E-10 |
|  | *LRRC32* | 0.60 | 1.79E-41 | 6.09E-39 |  | *NGLY1* | -0.32 | 2.17E-11 | 2.36E-10 |
|  | *CSDC2* | 0.60 | 1.81E-41 | 6.09E-39 |  | *CYCS* | -0.32 | 2.25E-11 | 2.44E-10 |
|  | *CORIN* | 0.60 | 2.09E-41 | 6.92E-39 |  | *GEN1* | -0.32 | 2.26E-11 | 2.46E-10 |
|  | *LUM* | 0.60 | 2.30E-41 | 7.52E-39 |  | *MTHFD1* | -0.32 | 2.73E-11 | 2.94E-10 |
|  | *FAM19A5* | 0.60 | 2.60E-41 | 8.36E-39 |  | *TCERG1* | -0.32 | 3.31E-11 | 3.54E-10 |
|  | *MOXD1* | 0.60 | 2.84E-41 | 8.99E-39 |  | *BRCC3* | -0.32 | 3.86E-11 | 4.08E-10 |
|  | *COL10A1* | 0.60 | 3.12E-41 | 9.72E-39 |  | *ESCO2* | -0.32 | 3.99E-11 | 4.21E-10 |
|  | *TIMP2* | 0.59 | 4.57E-41 | 1.40E-38 |  | *FAR1* | -0.32 | 4.00E-11 | 4.23E-10 |
|  | *ZFPM2* | 0.59 | 5.96E-41 | 1.80E-38 |  | *CNOT10* | -0.32 | 4.31E-11 | 4.53E-10 |
|  | *ST6GALNAC5* | 0.59 | 6.79E-41 | 2.02E-38 |  | *TTF2* | -0.32 | 4.32E-11 | 4.54E-10 |
|  | *EVC* | 0.59 | 8.65E-41 | 2.54E-38 |  | *RPS6KA1* | -0.32 | 4.80E-11 | 5.01E-10 |
|  | *CCDC80* | 0.59 | 1.13E-40 | 3.27E-38 |  | *VRK1* | -0.32 | 5.11E-11 | 5.32E-10 |
|  | *CERCAM* | 0.59 | 1.62E-40 | 4.61E-38 |  | *PTCD3* | -0.31 | 5.26E-11 | 5.47E-10 |
|  | *GEFT* | 0.59 | 1.75E-40 | 4.91E-38 |  | *PPIF* | -0.31 | 5.33E-11 | 5.54E-10 |
|  | *TMEM90B* | 0.59 | 2.75E-40 | 7.62E-38 |  | *MAP7* | -0.31 | 5.79E-11 | 5.99E-10 |
|  | *FBN1* | 0.59 | 3.62E-40 | 9.90E-38 |  | *ZGRF1* | -0.31 | 5.85E-11 | 6.06E-10 |
|  | *OLFML1* | 0.59 | 4.08E-40 | 1.10E-37 |  | *SLC37A1* | -0.31 | 6.71E-11 | 6.90E-10 |
|  | *PLXNA4* | 0.59 | 4.60E-40 | 1.22E-37 |  | *TPD52* | -0.31 | 6.92E-11 | 7.09E-10 |
|  | *GDF6* | 0.59 | 4.85E-40 | 1.27E-37 |  | *PPIL5* | -0.31 | 6.98E-11 | 7.15E-10 |
|  | *LMCD1* | 0.59 | 6.10E-40 | 1.58E-37 |  | *CDC7* | -0.31 | 7.10E-11 | 7.26E-10 |
|  | *SSC5D* | 0.59 | 7.33E-40 | 1.88E-37 |  | *GMDS* | -0.31 | 7.42E-11 | 7.55E-10 |
|  | *EFS* | 0.59 | 8.39E-40 | 2.12E-37 |  | *GPD2* | -0.31 | 7.65E-11 | 7.77E-10 |
|  | *THBS4* | 0.59 | 9.48E-40 | 2.37E-37 |  | *SASS6* | -0.31 | 7.69E-11 | 7.81E-10 |
|  | *MAP1A* | 0.59 | 1.08E-39 | 2.66E-37 |  | *RAVER2* | -0.31 | 7.73E-11 | 7.85E-10 |
|  | *DCN* | 0.58 | 1.85E-39 | 4.51E-37 |  | *RPL36A* | -0.31 | 8.24E-11 | 8.34E-10 |
|  | *MGP* | 0.58 | 2.35E-39 | 5.67E-37 |  | *IFRD2* | -0.31 | 8.33E-11 | 8.42E-10 |
|  | *VCAN* | 0.58 | 3.03E-39 | 7.22E-37 |  | *GMFB* | -0.31 | 8.72E-11 | 8.80E-10 |
|  | *FSTL1* | 0.58 | 3.73E-39 | 8.77E-37 |  | *MYO6* | -0.31 | 9.33E-11 | 9.37E-10 |
|  | *FAP* | 0.58 | 4.05E-39 | 9.42E-37 |  | *E2F2* | -0.31 | 9.85E-11 | 9.87E-10 |
|  | *CDO1* | 0.58 | 4.34E-39 | 9.97E-37 |  | *PDCD6IP* | -0.31 | 9.91E-11 | 9.92E-10 |
|  | *STON1* | 0.58 | 4.63E-39 | 1.05E-36 |  | *DLAT* | -0.31 | 1.04E-10 | 1.03E-09 |
|  | *DPYSL3* | 0.58 | 6.98E-39 | 1.57E-36 |  | *WDR43* | -0.31 | 1.05E-10 | 1.05E-09 |
|  | *OLFML2B* | 0.58 | 8.01E-39 | 1.78E-36 |  | *RCOR1* | -0.31 | 1.06E-10 | 1.05E-09 |
|  | *THY1* | 0.58 | 1.26E-38 | 2.78E-36 |  | *RBM25* | -0.31 | 1.22E-10 | 1.20E-09 |
|  | *PRDM6* | 0.58 | 1.28E-38 | 2.78E-36 |  | *MRRF* | -0.31 | 1.24E-10 | 1.22E-09 |
|  | *OLFML3* | 0.58 | 1.34E-38 | 2.88E-36 |  | *ETNK1* | -0.31 | 1.42E-10 | 1.39E-09 |
|  | *PTGER3* | 0.58 | 1.74E-38 | 3.71E-36 |  | *C5orf30* | -0.31 | 1.42E-10 | 1.39E-09 |
|  | *ARMH4* | 0.58 | 1.91E-38 | 4.03E-36 |  | *PLS1* | -0.31 | 1.43E-10 | 1.39E-09 |
|  | *MRC2* | 0.58 | 2.16E-38 | 4.50E-36 |  | *NT5C3* | -0.31 | 1.43E-10 | 1.40E-09 |
|  | *DOK5* | 0.58 | 6.60E-38 | 1.36E-35 |  | *CDC25A* | -0.31 | 1.55E-10 | 1.51E-09 |
|  | *TMEM119* | 0.57 | 8.68E-38 | 1.77E-35 |  | *NAA15* | -0.31 | 1.56E-10 | 1.51E-09 |
|  | *DKK2* | 0.57 | 1.69E-37 | 3.41E-35 |  | *NAA25* | -0.31 | 1.59E-10 | 1.54E-09 |
|  | *NOX4* | 0.57 | 1.72E-37 | 3.45E-35 |  | *IARS2* | -0.31 | 1.67E-10 | 1.62E-09 |

Table S2 Cox proportional hazard model analysis results in TIMER

|  | coef | HR | 95%CI | P-value |
| --- | --- | --- | --- | --- |
| Stage2 | 0.75 | 2.12 | 0.92-4.90 | 0.08 |
| Stage3 | 1.09 | 2.97 | 1.35-6.52 | 0.01 |
| Stage4 | 1.43 | 4.20 | 1.43-12.32 | 0.01 |
| Purity | -0.53 | 0.59 | 0.25-1.38 | 0.22 |
| Gendermale | 0.26 | 1.30 | 0.83-2.03 | 0.25 |
| RaceBlack | 0.51 | 1.66 | 0.66-4.19 | 0.29 |
| RaceWhite | 0.16 | 1.17 | 0.69-1.99 | 0.55 |
| Age | 0.04 | 1.04 | 1.02-1.06 | 0.001 |
| B cell | 5.09 | 161.62 | 1.15-22710.63 | 0.04 |
| CD8+T cell | -0.53 | 0.59 | 0.02-17.13 | 0.76 |
| CD4+T cell | -4.35 | 0.01 | 0.00-5.84 | 0.16 |
| Macrophage | 7.59 | 1969.97 | 21.93-176972.20 | 0.001 |
| Neutrophil | -2.05 | 0.13 | 0.00-503.61 | 0.63 |
| Dendritic | 0.42 | 1.52 | 0.06-41.51 | 0.81 |
| THBS1 | 0.00 | 1.00 | 0.78-1.28 | 0.99 |
| THBS2 | 0.02 | 1.02 | 0.81-1.28 | 0.86 |
| THBS3 | -0.06 | 0.94 | 0.62-1.44 | 0.79 |
| THBS4 | -0.01 | 0.99 | 0.87-1.13 | 0.90 |
| COMP | 0.06 | 1.07 | 0.95-1.20 | 0.30 |
