# Supplementary figures and images for: Diagnostic, Therapeutic, and Prognostic Value of the Thrombospondin Family in Gastric Cancer
Source: Front Mol Biosci. 2021 Apr 28;8:647095. doi: 10.3389/fmolb.2021.647095 (PMC8113821; doi:10.3389/fmolb.2021.647095)

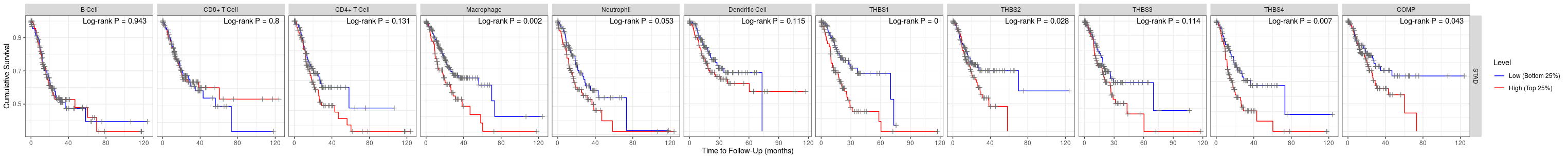

Supplement: Supplementary file 2 [file Image2.png]

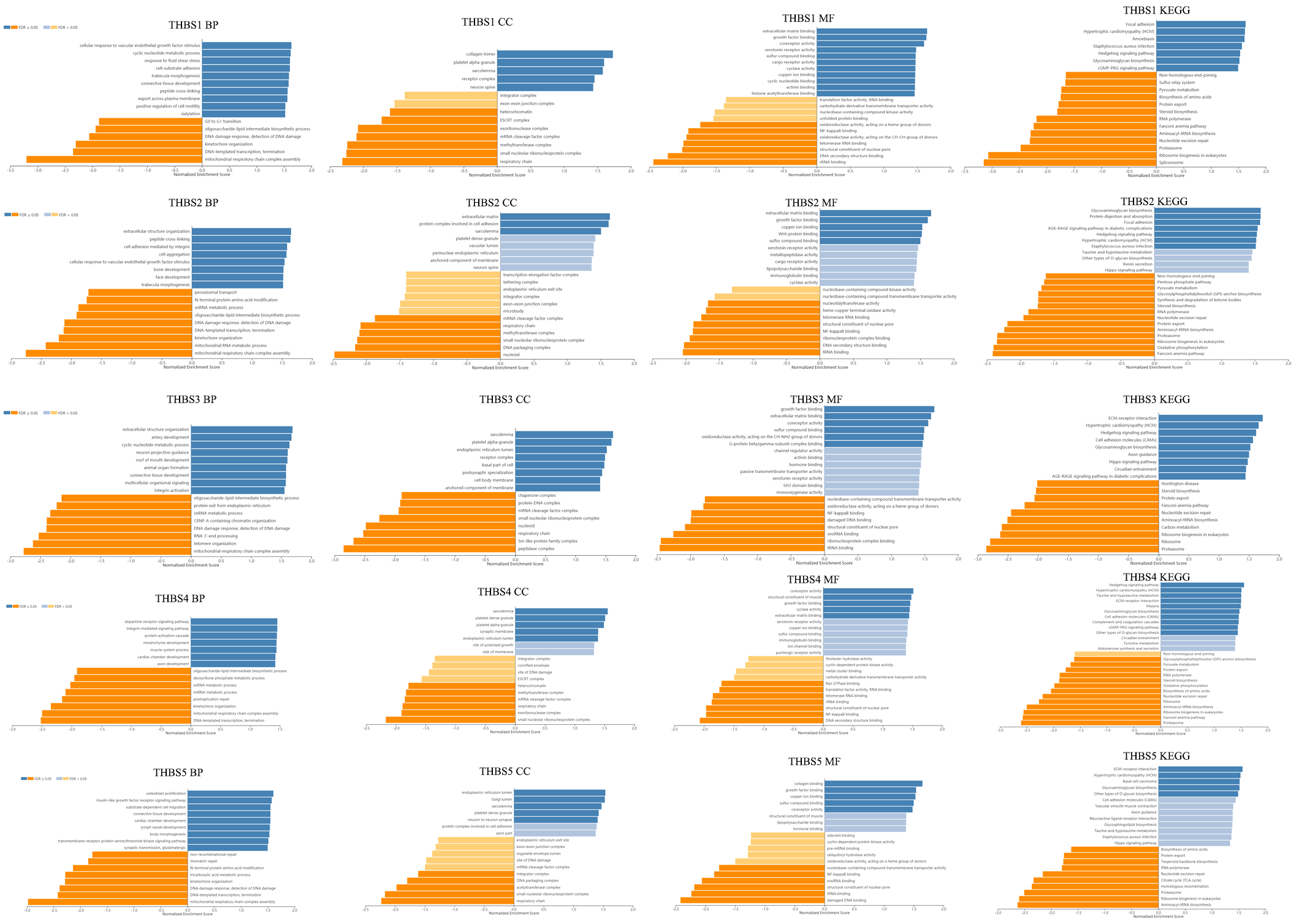

Supplement: Supplementary file 3 [file Image1.png]
