# Supplementary material for: Diagnostic, Therapeutic, and Prognostic Value of the Thrombospondin Family in Gastric Cancer
Source: Front Mol Biosci. 2021 Apr 28;8:647095. doi: 10.3389/fmolb.2021.647095 (PMC8113821; doi:10.3389/fmolb.2021.647095)
Supplement: Supplementary file 4 [file DataSheet1.docx]

Supplementary Figure Legends

Figure S1 Gene Ontology and Kyoto Encyclopedia of Genes and Genomes pathway analysis of the genes correlated with *THBSs* in gastric cancer (LinkedOmics)

Figure S2 The overall survival analysis of the patients with gastric cancer grouped by dominant immune cells infiltration levels and expression levels of *THBSs* (TIMER)
